# Apoptosis of *Mdm2*-deficient osteocytes enhances osteogenesis through TRPM8-enriched apoptotic vesicles

**DOI:** 10.1038/s41413-026-00570-0

**Published:** 2026-08-03

**Authors:** Xin Liu, Huiwen Zheng, Aochen Wang, Jianing Liu, Zhi Chen, Yiping Chen, Guobin Yang, Di Chen, Yufeng Zhang, Guohua Yuan

**Affiliations:** 1 State Key Laboratory of Oral & Maxillofacial Reconstruction and Regeneration, Key Laboratory of Oral Biomedicine Ministry of Education, Hubei Key Laboratory of Stomatology, School & Hospital of Stomatology, Wuhan University, Wuhan, China; 2 Frontier Science Center for Immunology and Metabolism, Wuhan University, Wuhan, China; 3 Hubei Provincial Key Laboratory of Developmentally Originated Disease, Wuhan, China; 4 Nanjing Stomatological Hospital, Affiliated Hospital of Medical School, Institute of Stomatology, Nanjing University, Nanjing, China; 5 Department of Paediatric Dentistry, School and Hospital of Stomatology, China Medical University, Shenyang, China; 6 Department of Cell and Molecular Biology, Tulane University, New Orleans, LA USA; 7 Faculty of Pharmaceutical Sciences, Department of Pharmacology, Shenzhen University of Advanced Technology, Shenzhen, China

**Keywords:** Bone, Homeostasis

## Abstract

During homeostasis, osteocyte apoptosis is typically associated with bone loss through enhanced osteoclast recruitment and bone resorption. However, whether apoptotic osteocytes also regulate bone formation remains elusive. Here we report that conditional deletion of *Mdm2*, an E3 ubiquitin ligase regulating cell survival, causes osteocyte apoptosis but paradoxically results in a marked increase in bone mass attributed to up-regulated osteogenic activity. Single-cell RNA sequencing reveals enhanced osteoblastic differentiation of bone marrow mesenchymal stem cells (BMSCs) in conditional knockout mice, with enrichment of cellular calcium related pathways. Mechanistically, apoptotic vesicles (apovs) from *Mdm2*-deleted osteocytes are engulfed by BMSCs. TRPM8, a calcium channel protein, is enriched in osteocyte-derived apovs and transported into BMSCs, thereby promoting osteoblastic differentiation. Additionally, pharmacological inhibition of TRPM8 attenuates the high bone mass phenotype in conditional knockout mice. Therefore, *Mdm2* deletion in osteocytes leads to osteocyte apoptosis, which enhances bone formation through communicating with BMSCs via TRPM8-enriched apovs. Our findings underscore the pivotal role of osteocytes in bone homeostasis and unveil a previously unrecognized mechanism whereby osteocyte apoptosis stimulates osteogenesis through affecting the fate of BMSCs in a TRPM8-mediated paracrine mechanism.

## Introduction

Bone homeostasis is maintained through the coordination of multiple cell types within the bone microenvironment, including osteoblastic lineage cells (OLCs) arising from bone marrow mesenchymal stem cells (BMSCs) and osteoclasts.^1,2^ Among these populations, osteocytes that comprise about 95% of the whole bone cells, function as key regulators in remodeling.^3,4^ Alive osteocytes possess extensive dendrites which form a complex communication network, enabling them to communicate with adjacent osteocytes or effector cells such as osteoblasts and osteoclasts, as well as to maintain the structure and functions of bone.^5–9^

Osteocytes are long-lived, terminally differentiated cells of the OLCs and are embedded within the mineralized bone matrix. Osteocyte death, particularly apoptosis, has long been associated with adverse effects on bone including loss of bone mass and deteriorated microarchitecture.^10–13^ Previous studies have shown that apoptotic osteocytes can regulate bone remodeling by recruiting osteoclasts and stimulating bone resorption, which is widely accepted.^11,13^ However, whether osteocyte apoptosis also exerts regulatory effects on bone formation and the underlying molecular mediators remain incompletely understood.

The E3 ubiquitin ligase MDM2 is a key regulator of various biological processes, including cell differentiation, proliferation, and apoptosis.^14–17^ Notably, apoptosis is primarily regulated through the targeting of the substrate protein p53.^18–23^ Although MDM2 has been studied in multiple aspects of bone biology, including proliferation and differentiation of pre-osteoblasts and pre-osteoclasts,^23,24^ its specific function in osteocyte survival and its potential involvement in osteocyte-mediated regulation of reshaping the bone microenvironment remain enigmatic.

In this study, we demonstrate that targeted deletion of *Mdm2* in osteocytes induces osteocyte apoptosis but paradoxically leads to a high bone mass phenotype. Mechanistically, apoptotic vesicles (apovs) secreted by *Mdm2* deficient osteocytes are enriched with the calcium channel protein TRPM8 and are subsequently engulfed by BMSCs, promoting osteoblastic differentiation. Additionally, pharmacological inhibition of TRPM8 attenuates the high bone mass phenotype in mice with targeted deletion of *Mdm2*. Our research reveals an unexpected mechanism by which apoptotic osteocytes regulate bone mass through vesicle-mediated communication with BMSCs. These findings uncover a novel osteocyte-BMSC signaling axis contributing to the maintenance of bone homeostasis.

## Results

### Deletion of *Mdm2* in the osteocytes leads to increased bone mass in mice

To verify MDM2 expression in osteocytes, immunostaining was performed. The results confirmed high expression of MDM2 in the cultured MLO-Y4 cells, an osteocyte-like cell line (Fig. S1a), as well as in osteocytes from the femurs of mice at 1 and 2 months (M) old (Fig. S1b).

**Fig. 1:**
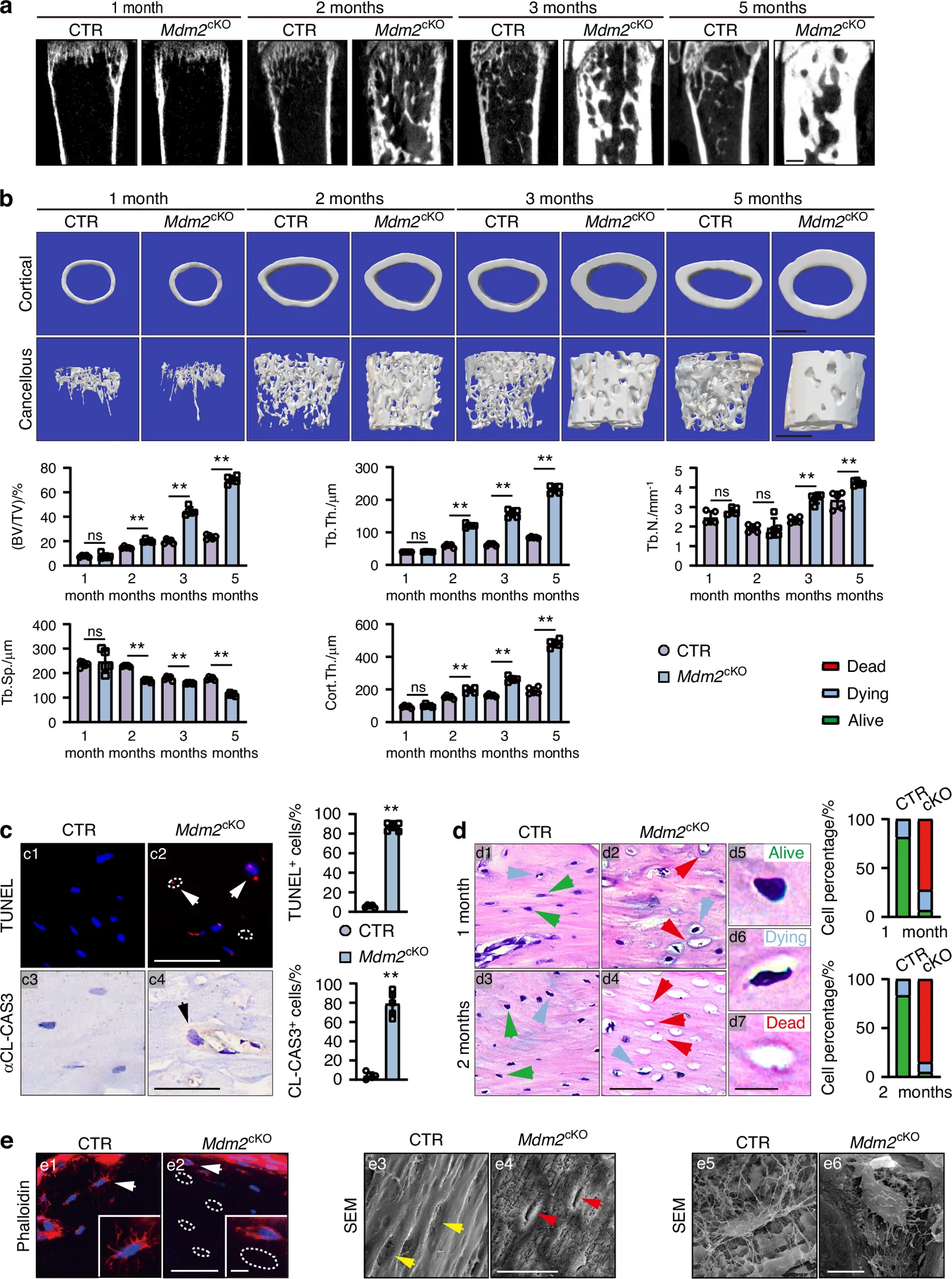
*Mdm2* deficiency in the osteocytes leads to high bone mass phenotype in mice as well as osteocyte apoptosis. **a** μ-CT images showing the femurs in *Mdm2* conditional knockout (*Mdm2*^cKO^) mice compared with control littermates at 1 month old (1 month), 2 months, 3 months and 5 months. **b** Three-dimensional reconstruction of μ-CT images in *Mdm2*^cKO^ mice compared with control littermates at 1 month, 2 months, 3 months and 5 months. Quantitative analysis of BV/TV, Tb.Th., Tb.N., Tb.Sp. and Cort.Th was presented (*n* = 5 males). **c** TUNEL staining and immunohistochemistry of CL-CAS3 in *Mdm2*^cKO^ mice at 1 month compared with control littermates. Histomorphometric quantification of positive cells was presented (*n* = 5 males). White arrows point to TUNEL positive osteocytes; Black arrow points to CL-CAS3 positive osteocytes; Dotted circles outline empty lacunae. **d** Alive (green arrows), dying (blue arrows) and dead (red arrows) osteocytes in *Mdm2*^cKO^ mice compared with control littermates at 1 month and 2 months by HE staining. Histomorphometric quantification of cell percentages was presented (*n* = 5 males). **e** Phalloidin staining of *Mdm2*^cKO^ mice at 1 month and scanning electron microscope (SEM) of *Mdm2*^cKO^ mice at 4 months showing the morphology of lacunae and osteocytes compared with respective control littermates. White, yellow and red arrows point to osteocytes, lacunae with osteocytes, and empty lacunae without osteocytes, respectively. Dotted circles outline empty lacunae. Data information: BV/TV, bone volume over total volume; Tb.Th. trabecular thickness, Tb.N. trabecular number, Tb.Sp. trabecular separation, Cort.Th. cortical thickness. Scale bars: 50 μm [**c**, **d** (c1–c4, d1–d4)], 10 μm [**d** (d5–d7), **e** (e5, e6)], 500 μm (**a**, **b**), 50 μm [**e** (e1–e4)]. Data are presented as mean ± SD (*n* = 5/group); **P < 0.01; 2-tailed unpaired Student’s *t* test (**b**, **c**)

To delineate the role of MDM2 in the osteocytes, we generated a conditional knockout mouse model by crossing *Mdm2*^flox/flox^ mice with *Dmp1*-Cre mice,^17^ and designated *Dmp1*-Cre; *Mdm2*^flox/flox^ mice as *Mdm2*^cKO^. Immunohistochemistry confirmed successful deletion of *Mdm2* in the osteocytes of *Mdm2*^cKO^ mice (Fig. S1b). Micro-computed tomography (μ-CT) showed comparable bone mass in the femurs of *Mdm2*^cKO^ mice with control mice at 1 month (Fig. 1a, b). However, the *Mdm2*^cKO^ mice exhibited markedly higher femoral bone density than control mice at 2 months, 3 months, and 5 months, as revealed by μ-CT and X-ray radiography (Fig. 1a; Fig. S1c). Three-dimensional reconstruction of μ-CT further showed that *Mdm2*^cKO^ mice at 2 months displayed markedly increased bone mass, which became more pronounced at 3 months and 5 months (Fig. 1b). The bone volume/tissue volume fraction (BV/TV) and trabecular thickness (Tb.Th.) at 2 months, 3 months and 5 months as well as the trabecular number (Tb.N.) at 3 months and 5 months were dramatically increased, while the trabecular separation (Tb.Sp.) at 2 months, 3 months and 5 months was decreased in *Mdm2*^cKO^ mice (Fig. 1b). Histological analyses by hematoxylin and eosin (HE) staining revealed that Tb.N., Tb.Th., and Cort.Th. were increased in *Mdm2*^cKO^ mice at 2 months, 3 months and 5 months while no obvious difference was found in mice at 1 month (Fig. S1d), consistent with the above μ-CT and X-ray results. Aligned with the femurs, bone mass in vertebral bone was dramatically increased in *Mdm2*^cKO^ mice compared with their control littermates at 3 months (Fig. S1e, f).

Consistent with the absence of significant change in bone mass at 1 month, femur and tibia lengths were also comparable between *Mdm2*^cKO^ mice and their control littermates at both 1 month and 2 months (Fig. S1g). The morphology of the femoral growth plate was also examined using HE staining. The results showed no significant differences in the heights of proliferative zone (PZ) and hypertrophic zone (HZ) between genotypes (Fig. S1h). Therefore, *Mdm2* deletion in osteocytes does not affect bone mass or longitudinal growth during developmental stage, and osteocyte-expressed MDM2 progressively regulates bone homeostasis in mice with age.

### Regulation of osteocyte apoptosis and bone homeostasis by the MDM2-p53 axis

To investigate the effects of *Mdm2* deletion on osteocytes, we conducted bulk RNA-seq on osteocytes harvested from mouse femurs. Gene Ontology (GO) analysis of differentially expressed genes (DEGs) revealed significant enrichment of apoptosis-related pathways, including “positive regulation of apoptotic signaling pathway” and “regulation of neuron apoptotic process” (Fig. S2a). These results indicated an association between *Mdm2* deletion and osteocyte apoptosis. To validate this possibility in vivo, Terminal deoxynucleotidyl transferase dUTP nick end labeling (TUNEL) staining and immunohistochemistry revealed an increased proportion of osteocytes positive for TUNEL or cleaved caspase-3 (CL-CAS3) (Fig. 1c). HE staining showed a massive increase in both dying and dead osteocytes in the femurs of *Mdm2*^cKO^ mice at 1 month and 2 months (Fig. 1d). Phalloidin staining and SEM showed that osteocytes in controls were rich in well-organized actin cytoskeleton, whereas those in *Mdm2*^cKO^ mice were shrunken and rounded, with fewer dendrites (Fig. 1e). HE staining, phalloidin staining, and SEM all showed an increase in empty lacunae in the femurs of *Mdm2*^cKO^ mice, whereas lacunae in control littermates were filled with normal osteocytes (Fig. 1d, e). These results suggest that *Mdm2* deletion induces osteocyte apoptosis in mice.

We also examined the role of MDM2 in osteocyte survival using the MLO-Y4 cells. The results showed that *Mdm2* knockdown induced apoptosis in MLO-Y4 cells as shown by Annexin V/PI flow cytometry, immunoblotting analysis, and TUNEL staining (Fig. S2b–e). p53, a well-known substrate of MDM2 that promotes apoptosis,^25^ mediated the osteocyte apoptosis induced by *Mdm2* knockdown, as concurrent *Tp53* knockdown rescued this effect. (Fig. S2b–e). Furthermore, immunofluorescent staining showed elevated p53 and p-p53 signals in the osteocytes of *Mdm2*^cKO^ mice (Fig. S3a), suggesting p53 accumulation and activation. We then performed an in vivo rescue experiment by crossing *Mdm2*^cKO^ mice with *Tp53*^flox/flox^ mice to generate *Dmp1-*Cre; *Mdm2*^flox/flox^; *Tp53*^flox/+^ mice (designated as *Mdm2*^cKO^; *Tp53*^fl/+^ mice). TUNEL staining indicated that the increased apoptosis of osteocytes in *Mdm2*^cKO^ mice was completely rescued by deletion of one allele of *Tp53* (Fig. S3b). Additionally, μ-CT experiments indicated that deletion of one allele of *Tp53* completely abolished the high bone mass phenotype of *Mdm2*^cKO^ mice (Fig. S3c–f). Quantitative analysis showed that BV/TV, Tb.Th., Tb.N., and Cort.Th. presented prominent reduction while Tb.Sp. was increased in *Mdm2*^cKO^; *Tp53*^fl/+^ mice compared to *Mdm2*^cKO^ mice, and no obvious difference was observed between *Mdm2*^cKO^; *Tp53*^fl/+^ and control mice (Fig. S3d, f). These results suggest that inhibition of osteocyte apoptosis rescues the high bone mass phenotype.

### Up-regulated osteogenic activity is responsible for the increased bone mass in *Mdm2*^cKO^ mice

Bone homeostasis is orchestrated by the functional activities of osteoclasts and osteoblasts.^1,2^ Anomalies in the functions of osteoblasts or osteoclasts can break the remodeling balance, leading to skeletal diseases such as osteoporosis, pathological fractures, bone deformity, and osteopetrosis.^26–28^ To reveal the mechanism underlying the high bone mass phenotype of *Mdm2*^cKO^ mice, we firstly analyzed bone resorption activity. Immunoblotting analysis showed increased protein levels of matrix metalloproteinase-9 (MMP9), nuclear factor of activated T-cells 1 (NFATC1) and tartrate-resistant acid phosphatase (TRAP) in the femurs of *Mdm2*^cKO^ mice at 1 month (Fig. S4a). ELISA assays revealed a markedly higher serum level of collagen type 1 cross-linked C-telopeptide (CTX-1), a bone resorption biomarker, in *Mdm2*^cKO^ mice (Fig. S4b). Consistently, analysis of TRAP staining images showed increased osteoclast surface/bone surface (Oc.S/BS) and osteoclast number/bone perimeter (Oc.N/BPm) in *Mdm2*^cKO^ mice (Fig. S4c). Immunohistochemistry showed increased numbers of NFATC1- and Cathepsin K (CK)-positive cells in *Mdm2*^cKO^ mice (Fig. S4d, e). These results reveal an apparent up-regulation of bone resorption in *Mdm2*^cKO^ mice compared to control mice, which can not explain the prominent high bone mass phenotype of the *Mdm2*^cKO^ mice.

**Fig. 2:**
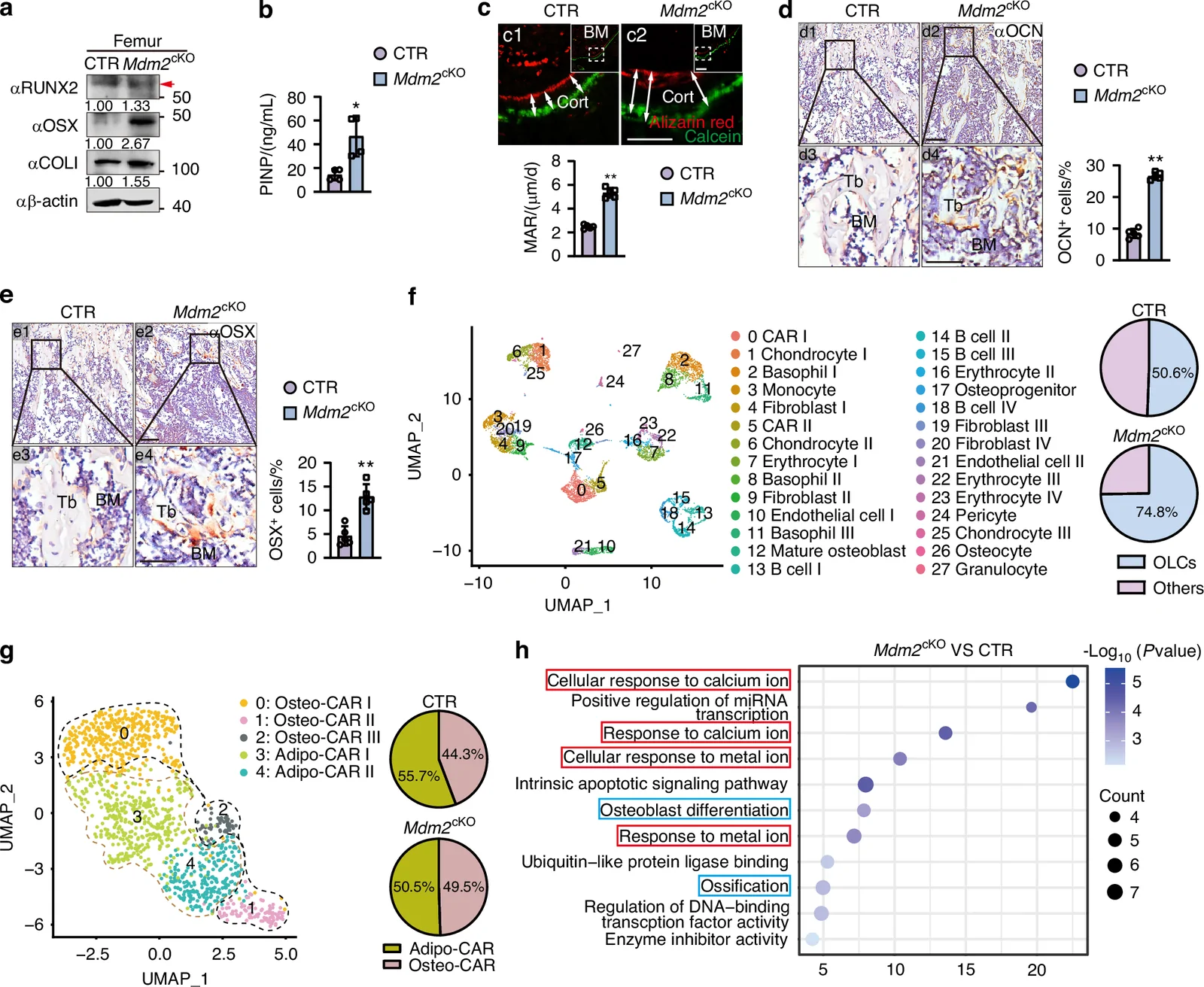
Osteoblastic differentiation and calcium ion related pathways in BMSCs are up-regulated in *Mdm2*^cKO^ mice. **a** Immunoblotting analysis showing the expression of osteogenesis related proteins RUNX2 (red arrow), OSX, and COLI in the femurs of *Mdm2*^cKO^ mice compared with control littermates at 1 month. β-actin served as the loading control. **b** PINP concentration in the serum of *Mdm2*^cKO^ mice compared with control littermates at 2 months by ELISA (*n* = 4 males). **c** Double labeling of Calcein and Alizarin red S (ARS) in the cortical bone of *Mdm2*^cKO^ mice and control littermates at 2 months. Calculation of the mineral apposition rate (MAR) was presented (*n* = 5 males). **d**, **e** Immunohistochemistry of OCN and OSX as well as histomorphometric quantification of the positive cells in *Mdm2*^cKO^ and control littermates at 2 months (*n* = 5 males). **f** UMAP plots showing the clustering of all sorted cells from bone marrow and matrix of *Mdm2*^cKO^ and control littermates at 2 months. The percentages of osteoblastic lineage cells (OLCs) in samples from *Mdm2*^cKO^ and control littermates was presented by pie charts. **g** UMAP plots of the scRNA-seq showing the BMSCs from *Mdm2*^cKO^ and control mice (including cluster 0 and 5 in Fig. 2f) with the subcluster 0, 3 and 4 as Osteo-CAR and the subcluster 1 and 2 as Adipo-CAR. Proportions of Osteo-CAR and Adipo-CAR in the BMSCs from *Mdm2*^cKO^ and control littermates were presented by pie charts. **h** GO analysis of Osteo-CAR showing enrichment of terms related to bone formation (blue rectangles) and calcium ion (red rectangles). Data information: BMSCs, bone marrow mesenchymal stem cells; Tb, trabecular bone; Cort, cortical bone; BM, bone marrow; CAR, *Cxcl12* abundant reticular cells. Scale bars: 100 μm [**c** (inserted magnification), **d** (d1, d2), **e** (e1, e2)], 50 μm **[c** (c1, c2), **d** (d3, d4), **e** (e3, e4)]. Data are presented as mean ± SD [*n* = 5/group (**c**, **d**, **e**); *n* = 4/group (**b**)]; *P < 0.05; **P < 0.01; 2-tailed unpaired Student’s *t* test (**b**, **c**, **d**, **e**)

We next investigated whether osteogenic activity is altered in *Mdm2*^cKO^ mice. Immunoblotting analysis showed up-regulated protein levels of osteogenic genes including Runt-related transcription factor 2 (RUNX2), Osterix (OSX) and collagen type I (COLI) in the femurs of *Mdm2*^cKO^ mice compared to the control mice at 1 month (Fig. 2a). ELISA assays showed that the serum level of procollagen type I N-terminal propeptide (PINP), a bone formation biomarker, was increased in *Mdm2*^cKO^ mice compared with their control littermates at 1 month (Fig. 2b). Calcein and Alizarin Red S (ARS) double labeling experiments, which measure the mineral apposition rate (MAR), indicated that MAR in *Mdm2*^cKO^ mice was dramatically increased compared to their control littermates (Fig. 2c). Immunohistochemistry of OSX and Osteocalcin (OCN) showed increased percentages of positive cells in *Mdm2*^cKO^ mice (Fig. 2d, e). In addition to endosteal osteogenesis, intramembranous bone formation driven by periosteal stem cells also contributes substantially to cortical bone mass.^29^ Therefore, we investigated whether periosteal bone formation was affected by loss of *Mdm2* in osteocytes. Calcein and ARS double labeling experiments indicated that periosteal MAR in *Mdm2*^cKO^ mice was also increased compared to their control littermates (Fig. S5a). Immunohistochemistry further showed increased percentages of OCN and OSX positive cells in the periosteum of *Mdm2*^cKO^ mice (Fig. S5b, c). These results collectively show that *Mdm2*^cKO^ mice exhibit apparent up-regulation of osteogenesis, which is consistent with the high bone mass phenotype.

### *Mdm2* deficiency promotes osteoblastic differentiation of BMSCs with enrichment of cellular calcium related pathways

To investigate the mechanisms underlying the phenotype of enhanced osteogenic activity and high bone mass in *Mdm2*^cKO^ mice, single-cell RNA sequencing (scRNA-seq) was performed to delineate the specific OLC subpopulation (Fig. S6a). In our experiments, 8 024 cells from control mice (*n* = 5) and 4 347 cells from *Mdm2*^cKO^ mice (*n* = 5) were captured. After uniform manifold approximation and projection (UMAP) clustering, cells were divided into 28 clusters (Fig. 2f) by different cell-specific markers (Fig. S6b). Among them, *Postn* characterized cluster 12 and 17 as osteoblasts, and *Dmp1* characterized cluster 26 as osteocytes (Fig. S6b, c). We sorted out clusters 12, 17, and 26, together with clusters 0 and 5, and collectively referred to them as OLCs. The results showed that the proportions of OLC clusters in *Mdm2*^cKO^ mice were increased compared to the control mice (Fig. 2f), consistent with our earlier results showing that osteogenic activity was up-regulated in *Mdm2*^cKO^ mice.

We defined clusters 0 and 5 as *Cxcl12*-abundant reticular (CAR) cells due to high expression levels of *Cxcl12* and other common stromal cell markers such as *Kitl* (Fig. S6c, d).^30–32^ The 1 206 cells from clusters 0 and 5 were integrated for subgroup classification into 5 subclusters based on the expression of osteogenic and adipogenic genes (Fig. 2g; Fig. S6e).^32–34^ The results showed that in *Mdm2*^cKO^ mice, the proportions of Osteo-CAR clusters were increased while the proportions of Adipo-CAR clusters were decreased (Fig. 2g). These results suggest that BMSCs in *Mdm2*^cKO^ mice display enhanced osteoblastic differentiation and impaired adipogenic commitment.

To reveal the underlying mechanisms along with enhanced osteoblastic differentiation of BMSCs in *Mdm2*^cKO^ mice, DEGs in the Osteo-CAR clusters were analyzed. GO analysis showed that DEGs were mainly associated with categories like “osteoblast differentiation” and “ossification”. Previous studies confirmed a critical role of calcium signaling in regulating osteoblastic differentiation,^35,36^ and we noticed several enriched categories were related to intracellular calcium such as “cellular response to calcium ion” and “cellular response to metal ion” (Fig. 2h). These results suggest enhanced osteoblastic differentiation of BMSCs is correlated to enrichment of cellular calcium ion related activities.

### *Mdm2* deficient osteocytes promote osteoblastic differentiation of BMSCs in a paracrine manner

**Fig. 3:**
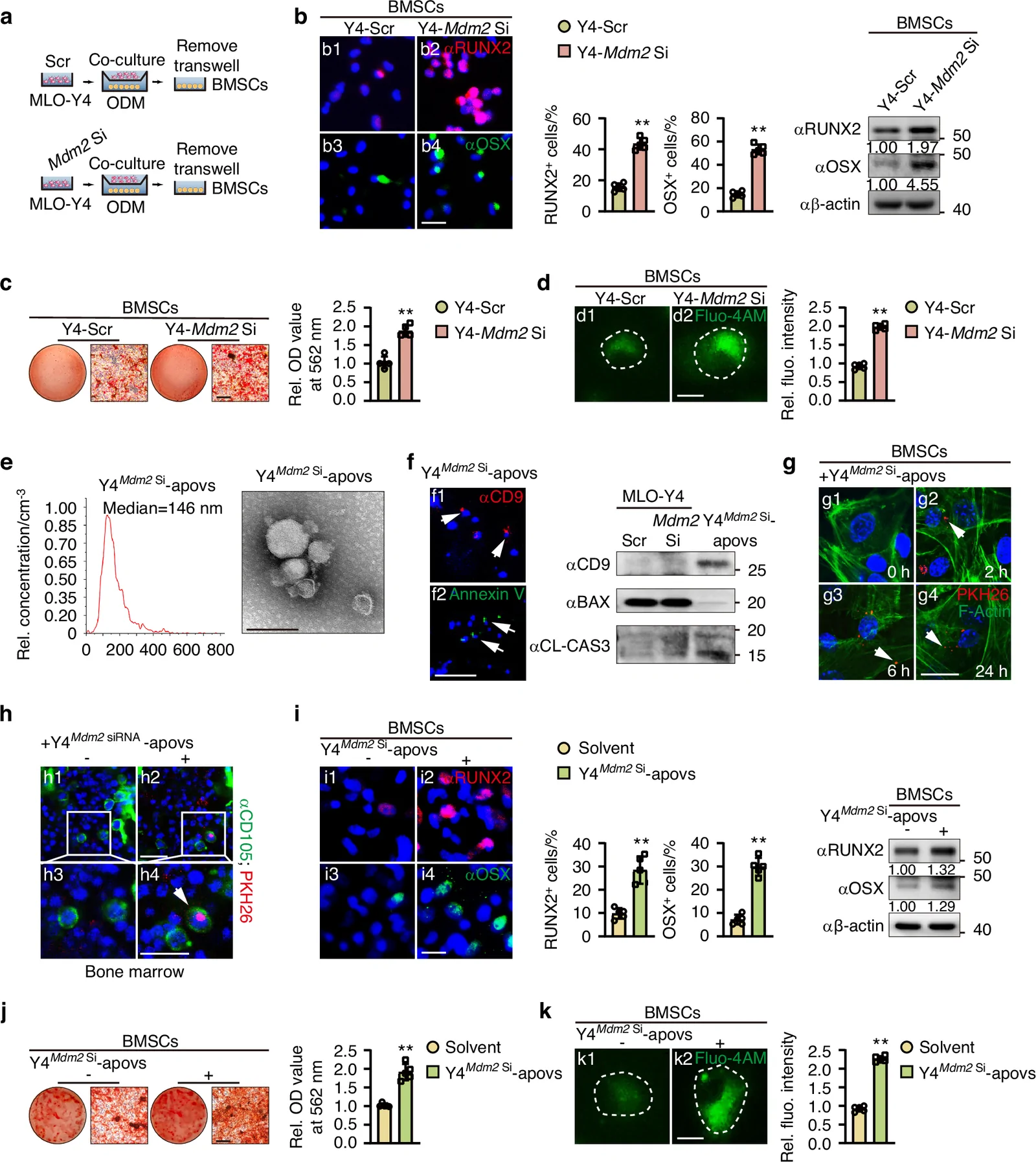
Apoptotic vesicles (apovs) derived from *Mdm2*-deficient osteocytes induce the osteoblastic differentiation of BMSCs. **a** Diagrammatic sketch of the co-culture strategy. **b** Immunofluorescence and immunoblotting analysis of RUNX2 and OSX in BMSCs after co-culture in ODM with MLO-Y4 cells transfected with *Mdm2* or scramble siRNA. Quantification of RUNX2 and OSX positive cells was presented. **c** ARS staining of BMSCs after co-culture in the ODM with MLO-Y4 cells transfected with *Mdm2* or scramble siRNA. Quantification of the eluted ARS dye was presented. **d** Fluo-4AM staining in BMSCs after co-culture with MLO-Y4 cells transfected with *Mdm2* or scramble siRNA. Quantification of Fluo-4AM fluorescence intensity was presented. **e** The size distribution of apovs shown by NTA and the morphology of apovs shown by TEM. **f** Immunofluorescence of Annexin V and CD9 in the apovs. Immunoblotting analysis of CD9, BAX and CL-CAS3 in the indicated MLO-Y4 cells and apovs. **g** Confocal microscopy images showing engulfment of apovs (PKH26 labeled) by BMSCs (F-actin) after co-culture for 0, 2, 6 and 24 h. **h** Confocal microscopy images showing engulfment of PKH26-labeled apovs by CD105-positive BMSCs 24 h after intravenous injection in vivo. **i** Immunofluorescence and immunoblotting analysis of RUNX2 and OSX in BMSCs cultured with ODM containing 1 μg/mL apovs or a solvent control. Quantification of RUNX2 and OSX positive cells was presented. **j** ARS staining of BMSCs cultured with 1 μg/mL apovs or a solvent control. Quantification of the eluted ARS dye was presented. **k** Fluo-4AM staining in BMSCs cultured with 1 μg/mL apovs or a solvent control. Quantification of Fluo-4AM fluorescence intensity was presented. Data information: White arrows point to the indicated apovs. NTA nanoparticle tracking analysis, TEM transmission electron microscopy, Scr Scramble siRNA; *Mdm2* Si; *Mdm2* siRNA; Y4^*Mdm2* Si^-apovs, apovs derived from MLO-Y4 cells with *Mdm2* knockdown; ODM, osteoblastic differentiation medium. Scare bars: 100 μm (**c**, **j**), 20 μm (**b**, **h**, **i**), 200 nm (**e**), 10 μm (**d**, **g, k**), 5 µm (**f**). Data are presented as mean ± SD (*n* = 5/group); **P < 0.01; 2-tailed unpaired Student’s *t* test (**b**, **c**, **d**, **i**, **j**, **k**)

To investigate whether the osteocytes deficient of *Mdm2* were responsible for the up-regulated osteoblastic differentiation of BMSCs, primary mouse BMSCs were co-cultured with MLO-Y4 cells transfected with scramble or *Mdm2* siRNA using an 8-μm transwell system in osteogenic differentiation medium (Fig. 3a). After co-culture with *Mdm2* knockdown MLO-Y4 cells, BMSCs showed increased expression of RUNX2 and OSX as assessed by immunofluorescent staining and immunoblotting analysis (Fig. 3b). ARS assays showed enhanced mineralization capacity of BMSCs co-cultured with *Mdm2* knockdown MLO-Y4 cells (Fig. 3c). Since DEGs in the BMSCs from *Mdm2*^cKO^ mice versus those from control mice were enriched in the calcium signaling pathways as described above, cultured BMSCs were stained with the calcium probe Fluo-4AM. The results showed that BMSCs co-cultured with *Mdm2* knockdown MLO-Y4 cells exhibited increased cytoplasmic fluorescence intensity compared to those co-cultured with control MLO-Y4 cells, suggesting elevated intracellular calcium levels (Fig. 3d).

To determine whether the osteogenic induction is specifically associated with apoptosis caused by *Mdm2* deficiency, we treated MLO-Y4 cells with a procaspase-3 activator, PAC-1, to induce cell apoptosis. BMSCs were then co-cultured with MLO-Y4 cells treated with scramble siRNA, PAC-1, or *Mdm2* siRNA (Fig. S7a). The results showed that compared with BMSCs co-cultured with *Mdm2* knockdown cells, those co-cultured with PAC-1 treated cells did not show increased levels of RUNX2 and OSX expression, enhanced mineralization capacity, or elevated intracellular calcium levels (Fig. S7b–d). Collectively, these results reveal that *Mdm2* knockdown MLO-Y4 cells enhance osteoblastic differentiation of BMSCs in a paracrine manner, and this effect specifically depends on *Mdm2* knockdown rather than general apoptosis.

Additionally, scRNA-seq results showed the decreased proportion of Adipo-CAR clusters. Consistently, BMSCs co-cultured with *Mdm2* knockdown MLO-Y4 cells under adipogenic induction showed reduced expression of *Pparγ* and *C/ebpα* (Fig. S8a) and decreased lipid droplet formation (Fig. S8b). These findings further support the osteogenic effect of *Mdm2* knockdown MLO-Y4 cells on BMSCs, potentially through a shift in lineage commitment away from adipogenesis.

### Apovs derived from *Mdm2*-deficient osteocytes promote osteoblastic differentiation of BMSCs

Apovs, a special type of extracellular vesicles secreted by apoptotic cells, have been demonstrated to participate in intercellular communications.^37^ To unveil whether apovs mediated the influence of osteocytes on BMSCs, the apovs released by MLO-Y4 cells with *Mdm2* knockdown were isolated as previously described.^38^ Nanoparticle tracking analysis (NTA) and transmission electron microscopy (TEM) analyses were conducted to reveal the size distribution and shapes of the apovs (Fig. 3e). Immunofluorescence and immunoblotting analysis confirmed the presence of the extracellular vesicle surface marker CD9 as well as apoptotic markers including Annexin V, BAX, and CL-CAS3 in the apovs (Fig. 3f). To determine whether the apovs were internalized by BMSCs, apovs were labeled by PKH26, a red cell membrane dye, and added to the culture medium of BMSCs. Confocal microscopy images demonstrated the presence of the PKH26-labeled apovs inside the BMSCs as early as 2 h post-treatment, with sustained signals at 6 and 24 h post-treatment (Fig. 3g). To further explain the engulfment in vivo, PKH26-labeled apovs were intravenously injected into mice, which were sacrificed 24 h later. Immunofluorescence of CD105 was performed to identify BMSCs. Confocal microscope images displayed clear co-localization of PKH26 signals with CD105-positive cells within the bone marrow niche (Fig. 3h). Thus, apovs from *Mdm2* knockdown MLO-Y4 cells were indeed engulfed by BMSCs.

To investigate the effects of the MLO-Y4 derived apovs on BMSCs, BMSCs were treated with the apovs in the osteogenic differentiation medium. Both cell immunofluorescence and immunoblotting analysis revealed enhanced expression of RUNX2 and OSX in the BMSCs cultured with apovs under osteogenic induction (Fig. 3i), together with ARS assays showing enhanced mineralization capacity (Fig. 3j). Fluo-4AM staining showed increased fluorescence intensity in the apovs treated BMSCs (Fig. 3k). Additionally, RT-qPCR results showed decreased expression of *Pparγ* and *C/ebpα*, and ORO staining revealed less intracellular lipid droplet formation of BMSCs treated with apovs derived from *Mdm2* knockdown MLO-Y4 cells (Fig. S8c, d). Collectively, these results indicate that MLO-Y4 cells with *Mdm2* knockdown determined the differentiation fate of BMSCs, promoting osteogenic differentiation while suppressing adipocytic differentiation via an apovs-mediated paracrine mechanism.

### TRPM8 and PIRT are enriched in apovs from *Mdm2*-deficient osteocytes

**Fig. 4:**
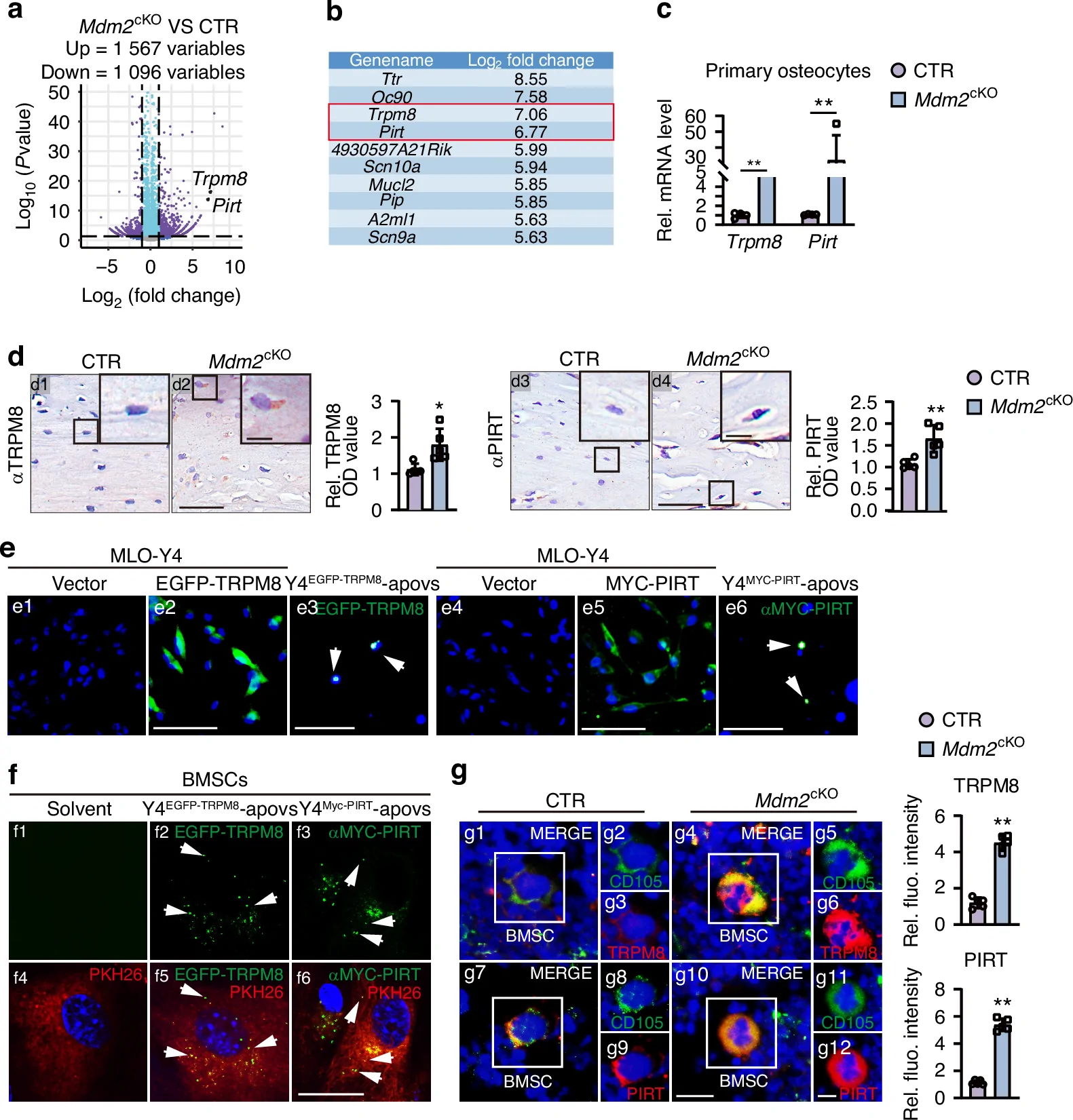
Up-regulated TRPM8 and PIRT in *Mdm2*-deficient osteocytes are enriched in apovs and transported into BMSCs. **a** Volcano plot showing DEGs in the osteocytes from *Mdm2*^cKO^ mice compared with those from control littermates at 3 months. **b** Table highlighting the top 10 up-regulated DEGs. **c**, **d** TRPM8 and PIRT levels in the osteocytes from *Mdm2*^cKO^ mice compared with control littermates at 3 months tested by RT-qPCR (*n* = 4 males) and immunohistochemistry. Histomorphometric quantification of the mean optical density was presented (*n* = 5 males). **e** The expression of EGFP-TRPM8 and MYC-PIRT in the indicated MLO-Y4 cells and apovs. White arrows point to the EGFP or MYC positive apovs. **f** Confocal microscopy images showing apovs enriched with EGFP-TRPM8 or MYC-PIRT are engulfed by the BMSCs. PKH26 was used to label BMSCs. White arrows point to the EGFP-TRPM8 or MYC-PIRT positive apovs that have been taken in by BMSCs. **g** Immunofluorescence showing TRPM8 and PIRT expression in CD105-positive BMSCs in *Mdm2*^cKO^ and control littermates. Histomorphometric quantification of the fluorescence intensities were presented (*n* = 5 males). Data information: DEGs, differentially expressed genes; Y4^EGFP-TRPM8^-apovs and Y4^MYC-PIRT^-apovs represent apovs derived from MLO-Y4 cells with EGFP-TRPM8 and MYC-PIRT overexpression respectively. Scale bars: 50 μm [**d**, **e** (e1, e2, e4, e5)], 10 μm [**d** (inserted magnifications), **f,** **g** (g2, g3, g5, g6, g8, g9, g11, g12)], 5 µm [**e** (e5, e6)], 20 μm **[g** (g1, g4, g7, g10)]. Data are presented as mean ± SD [*n* = 5/group (**d**, **g**); *n* = 4/group (**c**)]; *P < 0.05; **P < 0.01; 2-tailed unpaired Student’s *t* test (**c**, **d**, **g**)

Although apovs engulfment by BMSCs was evident, this uptake alone does not fully explain the observed osteogenic effects. To identify the specific components in apovs that are responsible for regulating the differentiation fate of BMSCs, we characterized the apov-producing *Mdm2*-deleted osteocytes by the bulk RNA-seq results of osteocytes. We identified 2663 DEGs in *Mdm2*-deficient versus control osteocytes (Fig. 4a). GO analysis of DEGs revealed extensive alterations in the ion transport process including “calcium ion import into cytosol” and “positive regulation of ion transmembrane transport” (Fig. S2a). Given the fact that scRNA-seq data showed enriched calcium related pathways in the BMSCs of *Mdm2*^cKO^ mice (Fig. 2h), we hypothesized that apovs secreted by *Mdm2*-deleted osteocytes transport calcium ions related factors into BMSCs to regulate their differentiation. Therefore, *Trpm8* and *Pirt*, two genes related to calcium ions among the top ten up-regulated DEGs in the osteocyte RNA-seq data, were considered as such candidate genes (Fig. 4b).

To test this hypothesis, expression alterations were first examined using RT-qPCR and immunohistochemistry, and the results confirmed that both mRNA and protein levels of TRPM8 and PIRT were increased in the osteocytes from *Mdm2*^cKO^ mice compared with control mice (Fig. 4c, d). Immunoblotting analysis confirmed that TRPM8 and PIRT were up-regulated in *Mdm2*-knockdown MLO-Y4 cells compared to control cells, and these proteins were enriched in the apovs derived from *Mdm2*-knockdown MLO-Y4 cells. However, when equal amounts of total protein were loaded, apovs derived from *Mdm2* knockdown MLO-Y4 cells displayed prominently higher levels of TRPM8 and PIRT proteins compared to apovs from PAC-1 treated MLO-Y4 cells (Fig. S9a). Additionally, when induced by the apoptosis inducer PAC-1, MLO-Y4 cells ectopically expressed EGFP-tagged TRPM8 or MYC-tagged PIRT, and the released apovs were positive for these ectopically expressed proteins (Fig. 4e). These results suggest that *Mdm2* deletion in the osteocytes leads to up-regulated TRPM8 and PIRT, which are enriched in the secreted apovs derived from apoptotic osteocytes.

Incubation of these apovs with BMSCs caused transfer of the EGFP-tagged TRPM8 and MYC-tagged PIRT into BMSCs (Fig. 4f). Additionally, in *Mdm2*^cKO^ mice, the protein levels of TRPM8 and PIRT were up-regulated in the CD105 marked BMSCs of the femurs as shown by immunofluorescent microscopy (Fig. 4g). In vitro studies also showed that BMSCs cultured with apovs derived from *Mdm2* knockdown MLO-Y4 cells exhibited elevated expression of TRPM8 and PIRT. In contrast, BMSCs cultured with apovs derived from PAC-1 treated MLO-Y4 cells did not show such upregulation (Fig. S9b). These results suggest that TRPM8 and PIRT are up-regulated specifically in *Mdm2*-deficient osteocytes, enriched in the secreted apovs, and then transported into BMSCs.

### TRPM8 in apovs induces osteoblastic differentiation of BMSCs

**Fig. 5:**
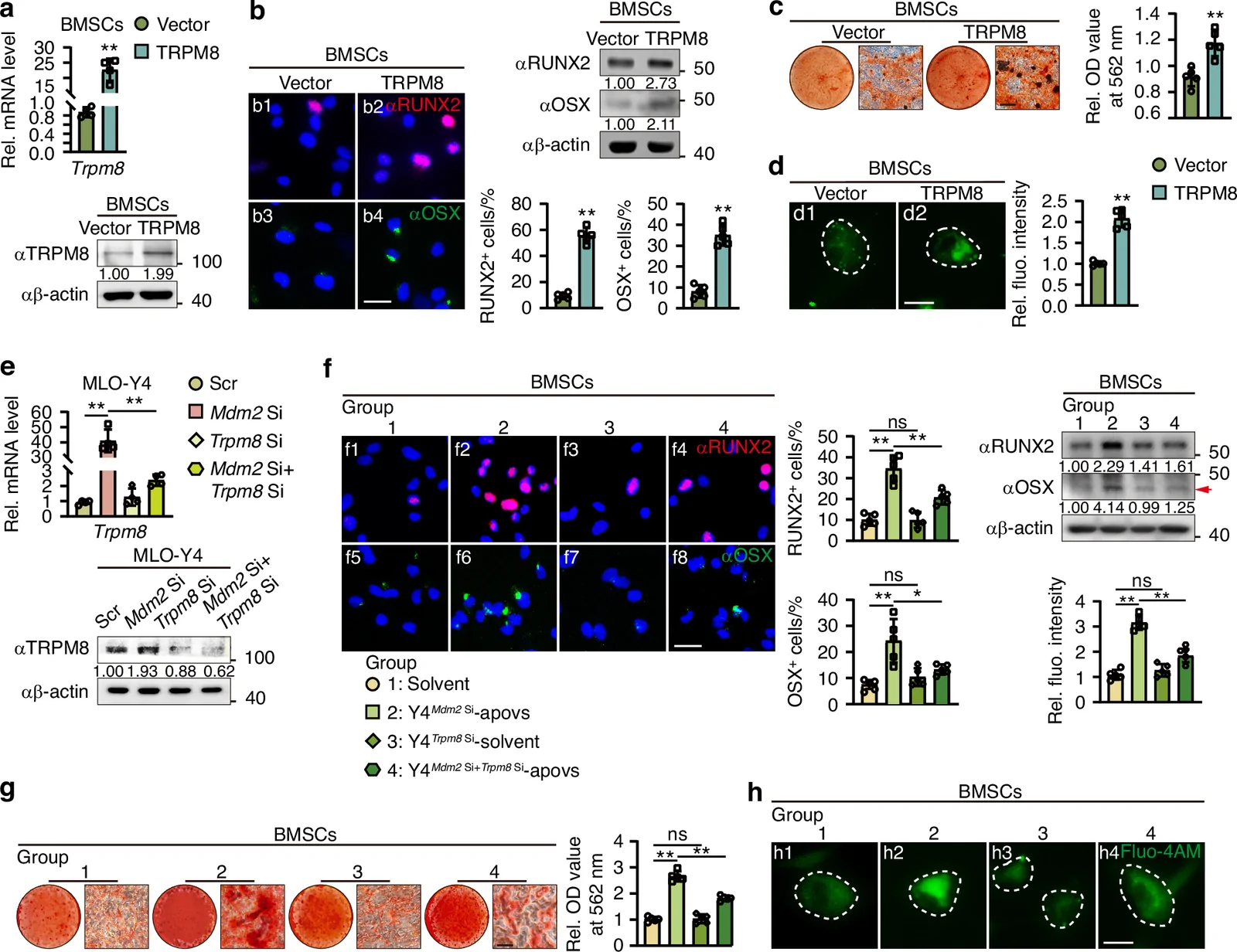
TRPM8 in apovs induces osteoblastic differentiation of BMSCs. **a** RT-qPCR and immunoblotting analysis showing successful overexpression of TRPM8 in BMSCs 48 h after transfection with overexpression plasmids. **b** Immunofluorescence and immunoblotting analysis of RUNX2 and OSX in the indicated BMSCs after culture in ODM for 4 days following transfection with overexpression plasmids. Quantification of RUNX2 and OSX positive cells was presented. **c** ARS staining of the indicated BMSCs after culture in the ODM for 2 weeks. Quantification of eluted ARS dye was presented. **d** Fluo-4AM staining of the indicated BMSCs 48 h after transfection with overexpression plasmids. Quantification of Fluo-4AM fluorescence intensity was presented. **e** RT-qPCR and immunoblotting analysis showing successful knockdown of *Mdm2* and *Trpm8* in MLO-Y4 cells 48 h after transfection with siRNAs. **f** Immunofluorescence and immunoblotting analysis of RUNX2 and OSX in BMSCs after culture in ODM supplemented with indicated apovs. The percentage of RUNX2 and OSX positive cells was presented. **g** ARS staining of BMSCs after culture in ODM supplemented with indicated apovs. Quantification of the eluted ARS dye was presented. **h** Fluo-4AM staining of BMSCs cultured with the indicated apovs. Quantification of the Fluo-4AM fluorescence intensity was presented. Data information: Scr, Scramble siRNA; *Mdm2* Si; *Mdm2* siRNA; *Trpm8* Si; *Trpm8* siRNA; Y4^*Mdm2* Si^-apovs, apovs derived from MLO-Y4 cells with *Mdm2* knockdown; Y4^*Trpm8* Si^-solvent, solvent from MLO-Y4 cells with *Trpm8* knockdown presenting no apoptosis; Y4^*Mdm2* Si+*Trpm8* Si^-apovs, apovs derived from MLO-Y4 cells with *Mdm2* and *Trpm8* knockdown; ODM, osteoblastic differentiation medium. Scale bars: 100 μm (**c**, **g**), 20 μm (**b**, **f**), 10 µm (**d**, **h**). Data are presented as mean ± SD [*n* = 4/group (**a**, **e**); *n* = 5/group (**b**, **c**, **d**, **f**, **g**, **h**)]; *P < 0.05; **P < 0.01; 2-tailed unpaired Student’s *t* test (**a**, **b**, **c**, **d**); ANOVA (**e**, **f**, **g**, **h**)

Since TRPM8 is the major calcium channel mediating ion influx and PIRT mainly functions as its enhancing factor,^39–41^ whether TRPM8 in apovs was the major factor mediating the osteoblastic differentiation of BMSCs was further tested. First, the effects of TRPM8 on BMSCs were tested. Overexpression of TRPM8 (Fig. 5a) resulted in up-regulated expression of osteoblast markers RUNX2 and OSX as assessed by immunoblotting analysis and immunofluorescent staining (Fig. 5b), enhanced mineralization capacities as measured by the ARS assays (Fig. 5c) and increased intracellular calcium levels as revealed by Fluo-4AM staining (Fig. 5d). These results suggest that TRPM8 can promote osteoblastic differentiation of BMSCs.

Second, apovs were collected from MLO-Y4 cells with separate or concurrent knockdown of *Mdm2* and *Trpm8* and added to the culture medium of BMSCs. Since direct knockdown of *Trpm8* did not induce cellular apoptosis, we employed solvent treatment as an alternative approach in the *Trpm8* knockdown group. The knockdown efficiencies were confirmed by RT-qPCR and immunoblotting (Fig. 5e). Compared with the BMSCs treated with apovs from *Mdm2* knockdown cells, the BMSCs treated with apovs from *Mdm2* and *Trpm8* double knockdown cells displayed decreased levels of RUNX2 and OSX as shown by immunoblotting analysis and immunofluorescent staining (Fig. 5f), diminished mineralization capacity as tested by ARS staining (Fig. 5g), and lower intracellular calcium levels revealed by Fluo-4AM staining (Fig. 5h). These results suggest that TRPM8 promotes the osteoblastic differentiation of BMSCs and mediates, at least partially, the up-regulated osteoblastic differentiation of BMSCs caused by apovs from *Mdm2*-deficient osteocytes.

Taken together, these results indicate that TRPM8 enriched in apovs is a key mediator of BMSC osteoblastic differentiation induced by *Mdm2* deficiency in osteocytes.

### TRPM8 mediates the high bone mass phenotype in *Mdm2*^cKO^ mice

**Fig. 6:**
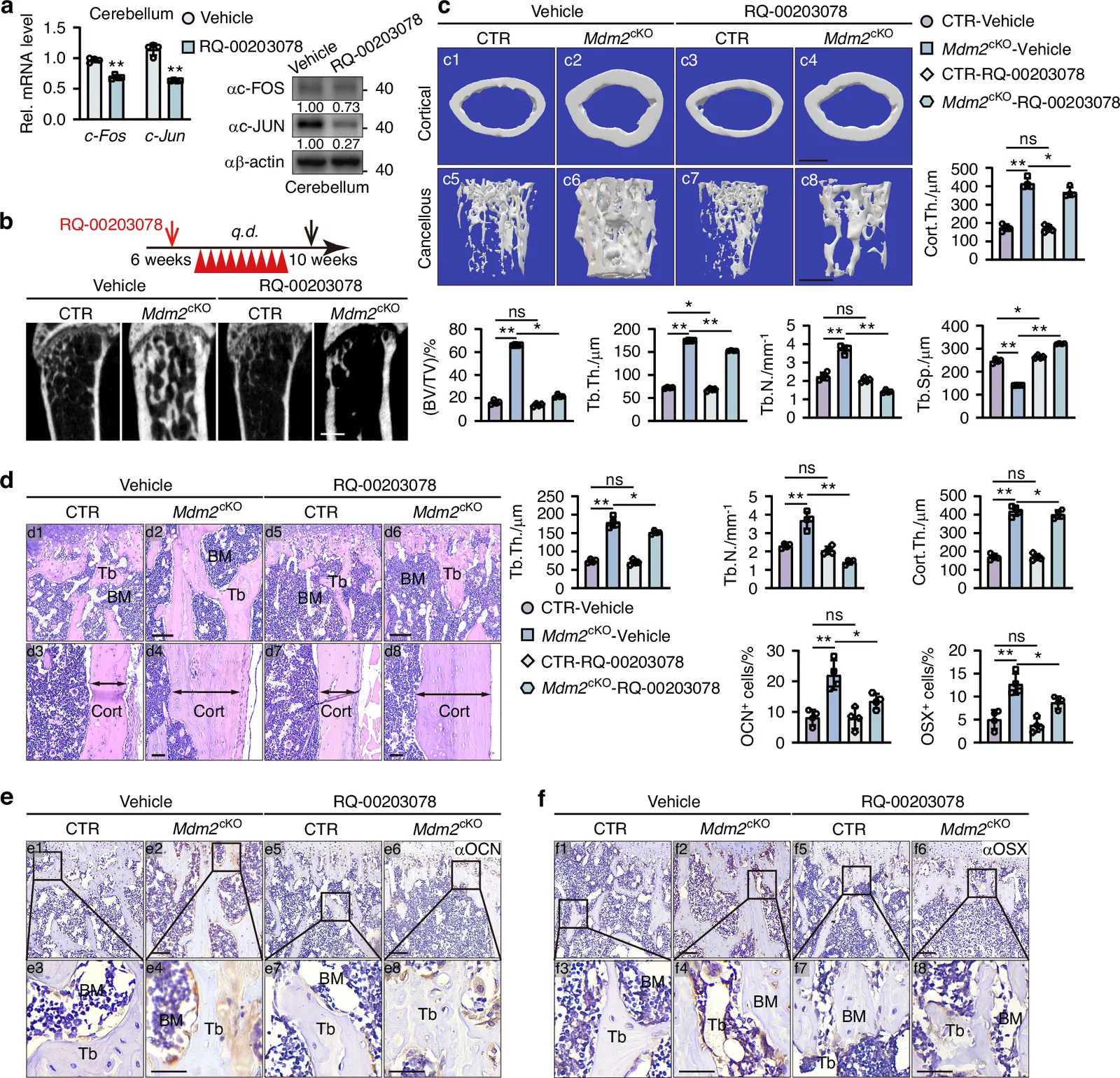
Pharmacological inhibition of TRPM8 attenuates the high bone mass phenotype in *Mdm2*^cKO^ mice. **a** RT-qPCR and immunoblotting analysis showing the expression of c-FOS and c-JUN in the cerebellum of mice treated with RQ-00203078 or vehicle control. **b** μ-CT images showing the femurs in *Mdm2*^cKO^ mice and control littermates treated with RQ-00203078 or vehicle control. **c** Three-dimensional reconstruction of μ-CT images of *Mdm2*^cKO^ mice and control littermates treated with RQ-00203078 or vehicle control. Quantitative analysis of BV/TV, Tb.Th., Tb.N., Tb.Sp. and Cort.Th. was presented. **d** HE staining of the femurs in *Mdm2*^cKO^ mice and control littermates treated with RQ-00203078 or vehicle control. Histomorphometric quantification of Tb.N., Tb.Th. and Cort.Th. was presented. **e**, **f** Immunohistochemistry of OCN and OSX as well as histomorphometric quantification of the positive cells in *Mdm2*^cKO^ mice and control littermates treated with RQ-00203078 or vehicle control. Data information: Tb, trabecular bone; Cort, cortical bone; BM, bone marrow. Scale bars: 500 μm (**b**, **c**); 200 μm [**d** (d1, d2, d5, d6)], 100 μm **[d** (d3, d4, d7, d8), **e** (e1, e2, e5, e6), **f** (f1, f2, f5, f6)], 50 μm [**e** (e3, e4, e7, e8), **f** (f3, f4, f7, f8)]. Data are presented as mean ± SD (*n* = 4 males/group); *P < 0.05, **P < 0.01; 2-tailed unpaired Student’s *t* test (**a**); ANOVA (**c**, **d**, **e**, **f**)

To determine whether TRPM8 serves as the primary mediator of MDM2-regulated high bone mass phenotype in vivo, *Mdm2*^cKO^ mice were treated with the TRPM8 antagonist RQ-00203078. RT-qPCR and immunofluorescence showed reduced expression of the early calcium signaling genes *c-Fos* and *c-Jun* (Fig. 6a), confirming the in vivo efficacy of the antagonist.

Mice at 6 weeks were treated with RQ-00203078 once daily until sacrifice at 10 weeks. μ-CT revealed a marked decrease in bone mass in *Mdm2*^cKO^ mice after RQ-00203078 treatment compared with those treated with vehicle control. In contrast, TRPM8 inhibition did not cause significant changes in bone mass in control littermates (Fig. 6b). Three-dimensional reconstruction of μ-CT, quantitative analysis, and HE staining results further showed that the high bone mass in both cancellous and cortical bone of *Mdm2*^cKO^ mice was significantly reduced following RQ-00203078 treatment compared with vehicle controls (Fig. 6c, d). In contrast. TRPM8 inhibition only slightly affected the bone mass in the control littermates (Fig. 6c, d). Immunohistochemistry of OSX and OCN showed decreased percentages of positive cells in RQ-00203078 treated *Mdm2*^cKO^ mice compared to those with vehicle treatment (Fig. 6e, f).

Together, these results show that pharmacological inhibition of TRPM8 markedly attenuates the high bone mass phenotype in *Mdm2*^cKO^ mice, indicating that TRPM8 is the critical mediator of bone formation driven by *Mdm2* deficient osteocytes.

### MDM2-p53 axis negatively regulates TRPM8 post-translationally and transcriptionally

Given the negative correlation between MDM2 and TRPM8 levels in the osteocytes, we raised the question how MDM2 regulates the level of TRPM8. In situ proximity ligation assays (PLA) indicated that MDM2 and TRPM8 are in close proximity in MLO-Y4 cells (Fig. S10a). Their interaction was further confirmed by transient transfection and co-IP experiments (Fig. S10b). Co-IP and ubiquitination assays indicated that MDM2 catalyzed polyubiquitination of TRPM8 in HEK293T cells (Fig. S10c). Cycloheximide (CHX) chase assay indicated that MDM2 reduced the half-life of TRPM8 (Fig. S10d). Additionally, MDM2-mediated down-regulation of TRPM8 was inhibited by the proteasomal inhibitor MG132 but not by the lysosomal inhibitor chloroquine (Fig. S10e), suggesting that MDM2 down-regulates TRPM8 by the ubiquitination-proteasomal pathway.

Interestingly, increased *Trpm8* mRNA level was also observed in the *Mdm2-*deleted osteocytes (Fig. 4b). Since p53, an extensively studied substrate of MDM2, functions as a vital transcriptional factor in biological processes,^42^ and was reported to promote the expression of human TRPM8,^43^ we attempted to address whether MDM2 regulates TRPM8 mRNA level through a p53-dependent mechanism. Reporter assays indicated that overexpression of MDM2 inhibited *Trpm8* promoter activity, while overexpression of p53 activated *Trpm8* promoter activity (Fig. S10f). RT-qPCR results showed that *Mdm2* knockdown increased *Trpm8* mRNA level, which was reversed by further knockdown of *Tp53* (Fig. S10g). These results suggest that MDM2 negatively regulates TRPM8 by directly promoting its ubiquitination and proteasomal degradation and indirectly antagonizing p53-mediated transcription of *Trpm8*.

## Discussion

Osteocytes are terminally differentiated cells, and their survival is indispensable for bone homeostasis. Despite extensive studies in other cell types, the functions of MDM2 in osteocytes and its contribution to bone mass through osteocyte apoptosis remain understudied. In this study, we identify that deletion of *Mdm2* in osteocytes leads to osteocyte apoptosis, accompanied by enhanced bone formation through apov-mediated TRPM8 signaling. These findings reveal a previously unrecognized osteocyte–BMSC crosstalk regulating bone remodeling.

Accumulating evidence suggests that osteocytes in abnormal biological states can alter bone mass. Osteocyte apoptosis mice generated using *Dmp1*-Cre; *iDTR* display thinner cortical bone, reduced trabecular bone mass and decreased flexural resistance,^10^ indicating detrimental effects of osteocyte apoptosis upon bone mass and tissue structure. Similarly, mouse models with osteocyte senescence or necrosis also exhibit bone loss and skeletal deterioration.^44–46^ These findings support the notion that osteocyte loss is generally detrimental to bone homeostasis, leading to bone loss. However, our study reveals an opposing outcome, in which apoptosis in *Mdm2* deficient osteocytes leads to a high bone mass phenotype. These results suggest that osteocyte apoptosis in our specific model triggers a distinct biological response during bone remodeling.

In *Mdm2*^cKO^ mice, we also observed increased bone resorption, resembling the phenotype reported in most osteocyte ablation models.^11,46^ However, the increased bone mass in *Mdm2*^cKO^ mice indicates that apart from the impact on osteoclastogenesis, more compensatory osteogenesis might be induced. Indeed, we observed markedly augmented osteogenesis in *Mdm2*^cKO^ mice, which effectively overwhelms the increased osteoclastogenesis and results in the high bone mass phenotype. Therefore, the imbalance between increased bone resorption and enhanced bone formation helps explain the discrepancy between our results and previous osteocyte ablation studies. Additionally, our study presents a phenotype similar to that observed in *Fgfr1*/*Fgfr2* double-conditional knockout mice.^47^ Collectively, our findings expand the current understanding of osteocyte apoptosis in skeletal homeostasis.

Consistently, scRNA-seq analysis revealed a significant expansion of the OLCs accompanied by enrichment of calcium signaling pathways. This observation drew our attention to BMSCs, which serve as the progenitor source of OLCs.^48,49^ BMSCs possess bipotent differentiation potential toward osteoblastic or adipocytic lineages. Therefore, promoting this process toward osteoblastic differentiation is particularly important for maintaining and expanding the OLC population.^33,34,50^ Notably, our results showed a differentiation bias toward osteoblasts in BMSCs derived from *Mdm2*^cKO^ mice.

In addition to osteocytes, we found that *Dmp1*-Cre also targets osteoblasts, as demonstrated in *Dmp1*-Cre; *R26R*^mTmG^ mice (Fig. S11a–c).^51,52^ Subsequent experiments indicated that *Mdm2* knockdown in the osteoblast cell line inhibited osteogenic capacity (Fig. S11d, e), which is consistent with the impaired osteogenesis observed in *Mdm2* deleted pre-osteoblasts using the *Col3.6*-Cre driver in a previous study.^24,53^ However, *Mdm2* deletion in *Dmp1*-Cre targeted osteocytes enhanced osteogenesis by markedly promoting osteoblastic differentiation of BMSCs, leading to a substantial increase in the osteoblast pool. This expansion outweighs the diminished osteogenesis observed in individual *Dmp1*-Cre-targeted osteoblasts. These findings highlight the stage-specific functions of MDM2 in different OLCs.

During apoptosis, cells secrete apovs ranging from nanoscale to microscale sizes.^37,54^ Functioning as important mediators, apovs participate in regulating bone mass through intricate intercellular communications between cells in the bone microenvironment.^55–58^ Co-culture experiments uncovered a functional crosstalk between *Mdm2* deleted apoptotic osteocytes and BMSCs, prompting us to investigate whether apovs mediate this interaction. We found that BMSCs could indeed engulf *Mdm2* deleted osteocyte-derived apovs and exhibited promoted osteogenesis accompanied by elevated intracellular calcium levels. These findings indicate that apoptotic vesicles are a novel mediator of communication linking apoptotic osteocytes to osteoblastic differentiation in BMSCs. However, apov uptake alone does not fully explain the mechanism underlying this osteogenic induction.

Different forms of osteocyte death lead to the accumulation of several bioactive factors including SASP components, DAMPs, and osteoclastogenic regulators such as RANKL, which modulate osteoclast activity.^10,44,46^ Analogously, apovs selectively encapsulate and transport bioactive cargo including proteins, lipids, and nucleic acids, which can be internalized by recipient cells to exert regulatory functions.^59,60^ This prompted us to search for the key components mediating the osteogenic effect of apovs from *Mdm2* deleted apoptotic osteocytes. *Trpm8*, with massive augmentation in *Mdm2* deleted osteocytes, was considered as a strong candidate, since TRPM8 activation in BMSCs mediates calcium influx to potentiate osteoblastic differentiation.^61^ We confirmed that TRPM8 is enriched in apovs derived from *Mdm2* deficient osteocytes and is subsequently delivered to recipient BMSCs. Functional gain-and loss-of-function experiments reveal that *Trpm8* overexpression enhanced osteogenesis in BMSCs while *Trpm8* knockdown in apovs significantly attenuated this effect. Together, these findings highlight TRPM8 as a critical cargo mediating apov-induced osteoblastic differentiation of BMSCs.

To further determine whether TRPM8 contributes to the high bone mass phenotype in *Mdm2*^cKO^ mice, we pharmacologically inhibited TRPM8 using a selective antagonist. The results showed that TRPM8 inhibition markedly reduced bone mass in *Mdm2*^cKO^ mice, providing in vivo evidence for its functional role. This effect was particularly evident in trabecular bone where BMSCs are the main source of new osteoblasts. Our results therefore highlight the ion channel protein TRPM8 as a key mediator for the effects of *Mdm2* deficient osteocytes on bone mass. These findings suggest that TRPM8 signaling may represent a previously unrecognized regulator of osteogenic induction.

TRPM8 in human bone tissues remains at a low level to maintain appropriate function.^62^ In contrast, our results further revealed that MDM2 negatively regulates TRPM8 at both protein level and the mRNA level. This helps explain the enrichment of TRPM8 in apovs derived from *Mdm2* deficient osteocytes.

In conclusion, our study innovatively unveils that apoptosis of *Mdm2* deleted osteocyte functions as an active regulator of osteogenesis through TRPM8 enriched apovs. This finding helps expand the role of apoptotic osteocytes apart from osteoclastogenesis, which inspires us to consider that different forms of osteocyte apoptosis may lead to completely different biological outcomes.

### Limitations of the study

While rescue experiments revealed that TRPM8 was a key factor in the apovs from the osteocytes lacking MDM2 to drive the osteoblastic differentiation of BMSCs, the partial rescue effects implied that TRPM8, while critical, was not the sole factor in the apovs for inducing the osteoblastic differentiation of BMSCs. Additional unidentified osteogenic factors may exist in the apovs, which orchestrate with TRPM8 and contribute to the phenotype of *Mdm2*^cKO^ mice.

## Methods and materials

### Mice

All mice were maintained on a C57BL/6 background, and experiments were conducted in accordance with the Animal Welfare and Ethics Committee of the School and Hospital of Stomatology at Wuhan University (S07921070D). Mice were housed 4–6 per cage under pathogen-free conditions at 20-24 °C and exposed to a 12-h/12-h light/dark cycle. *Mdm2*^cKO^ mice were generated as previously described.^17^ *Tp53*^flox/flox^ mice were generously provided by Prof. Bo Zhong (Wuhan University, Wuhan, China) and bred with *Mdm2*^cKO^ mice to generate *Mdm2*^cKO^; *Tp53* ^flox/+^ mice. *R26R*^mTmG^ mice (Shulaibao Biotechnology) were bred with *Dmp1*-Cre mice to generate *Dmp1*-Cre; *R26R*^mTmG^ mice. Details of the genotyping sequences are provided in Table S1. Both male and female animals were examined, and similar findings were obtained for both sexes. The *Mdm2*^flox/flox^ littermates without any bone phenotype were used as control mice. Data from 5 male mice in each experimental or control group are shown.

### TRPM8 antagonist administration

RQ-00203078 was dissolved in PBS containing 5.0% dimethyl sulfoxide at a concentration of 2.5 μg/μL. *Mdm2*^cKO^ mice at 6 weeks were intraperitoneally injected daily with RQ-00203078 solution or solvent control until sacrifice at 10 weeks. Femurs were harvested for subsequent analysis.

### Cell culture and transfection

The murine osteocyte cell line MLO-Y4 and the pre-osteoblast cell line MC3T3-E1 were purchased from Pricella. Primary mouse BMSCs were isolated from murine femurs and tibias using a standardized protocol as previously described. MLO-Y4 cells, MC3T3-E1 cells, and BMSCs were cultured in α-minimal essential medium (α-MEM; Hyclone) supplemented with 10% fetal bovine serum (FBS; Gibco) and 100 U/mL penicillin/streptomycin (P/S; Hyclone). HEK293T cells were cultured in Dulbecco’s modified Eagle medium (DMEM; Hyclone) supplemented with 10% fetal bovine serum (FBS; TBD15HT) and 100 U/mL P/S. BMSCs were cultured in differentiation medium after reaching 100% confluence to exclude potential effects caused by proliferation. For osteogenic induction, BMSCs were cultured in osteoblastic differentiation medium containing 10 mmol/L sodium β-glycerophosphate (Sigma-Aldrich), 50 µg/mL ascorbic acid (Sigma-Aldrich), and 10 nmol/L dexamethasone (Sigma-Aldrich) for 4–14 days. For adipogenic induction, BMSCs were cultured in adipocytic differentiation medium containing 500 μmol/L isobutylmethylxanthine (Sigma-Aldrich), 200 μmol/L indomethacin (Sigma-Aldrich), 1 μmol/L dexamethasone, and 0.5 μg/mL insulin (Sigma-Aldrich) for 10–14 days. All cultures were incubated at 37 °C with 5% CO₂ in a humidified environment to maintain optimal growth conditions.

The siRNAs targeting *Mdm2*, *Tp53* and *Trpm8* were designed and synthesized by GenePharma. Details of the siRNA sequences are listed in Table S2. Plasmids (*pCMV*-*Flag*-*Mdm2* and *pCMV*-*HA*-*Ub*) were previously described.^16^ *pFROG*-*Egfp*-*Trpm8* plasmids were generously provided by Prof. Jing Yao (Wuhan University, Wuhan, China). *pCMV*-*Myc*-*Pirt*, *pCMV-Tp53*, and *pGL3*-*Trpm8*-promoter plasmids were purchased from Origene. For transfection, cells were cultured to 80% confluence and then transfected using Lipofectamine 3000 reagent (Invitrogen) according to the manufacturer’s instructions.

For co-culture experiments, BMSCs were seeded in the lower chamber while MLO-Y4 cells were seeded at a quantitative proportion of 1:10 to BMSCs in the upper chamber of a Transwell system with an 8-μm pore polycarbonate membrane (Corning). MLO-Y4 cells were transfected with scramble or *Mdm2* siRNA. Scramble siRNA transfected MLO-Y4 cells were treated with or without PAC-1 (100 μmol/L, MedChemExpress).

### Bone preparation and tissue section

Mice were euthanized. The femurs as well as the third and fifth lumbar vertebrae (L3 and L5) were dissected. After removal of muscles, the femurs were fixed in ice-cold 4% paraformaldehyde (PFA, Servicebio) for 8 h. The femurs were then rinsed with phosphate-buffered saline (PBS, pH 7.4) at 4 °C overnight to guarantee that the remaining PFA was completely removed. Decalcification was then carried out using 0.5 mol/L EDTA at 4 °C under gentle agitation.

For paraffin sections, the femurs were dehydrated, embedded in paraffin and then sectioned into 5-μm slices. Before staining, paraffin sections were dewaxed and rehydrated.

For frozen sections, the femurs were embedded in OCT compound (Sakura), and sectioned at 10 μm on a cryostat (Leica, CM1950). Before staining, the mountant was washed off with running PBS.

### Bone histomorphometry assessment

For X-ray examination, the femurs were captured using Bruker In-Vivo DXS/F/FX PRO system with an exposure time of 240 s.

For micro-CT, the femurs were scanned using a Micro-CT imaging system (Perkin Elmer, Quantum GX2). The three-dimensional images were reconstructed using Mimics Research 20.0 Software. A volume of interest in the metaphyseal secondary spongiosa, starting 0.5 mm below the growth plate and extending 2.0 mm, was selected for analysis. CT Analysis Software was applied to calculate the following bone parameters: BV/TV, Tb.Th., Tb.N., Tb.Sp., and Cort.Th..

For SEM, after complete removal of adherent tissues, the femurs were chemically decellularized by 3% hydrogen peroxide solution (Sigma-Aldrich) at room temperature for 48 h and then rinsed extensively with distilled water until no residual oxidant was detectable (confirmed by peroxide test strips). The specimens were then placed in a desiccator (Nalgene) to dry out. Dry samples were mounted on aluminum stubs (Ted Pell) and sputter-coated (Emitech SC500) with a 60:40 gold-palladium alloy (Ted Pell) at 20 mA for 120 s to achieve a 15-nm conductive layer, as calibrated by quartz crystal microbalance. SEM images were captured using Scanning electron microscope (TESCAN, VEGR3LMU) at 10-30 kV.

### Calcein and ARS labeling

For calculation of MAR, 8-week-old mice were injected with calcein (20 mg/kg, Sigma-Aldrich) followed by administration of ARS (40 mg/kg, Sigma-Aldrich) 5 days later and sacrifice 7 days later. The femurs were collected, fixed, dehydrated and embedded in polymethyl methacrylate (PMMA) by Servicebio company. Five-μm sections were prepared using a hard tissue slicer and captured with the microscope (Leica DM4000B).

### HE staining, IHC staining and TRAP staining

For HE staining, rehydrated paraffin-embedded sections were immersed in hematoxylin solution (Servicebio) for 10 s and in eosin solution (Servicebio) for 90 s.

For IHC, antigens were retrieved using a protease-based method (MaxVision kit). Endogenous peroxidase activity was blocked by H_2_O_2_ solution from HRP Polymer anti-Rabbit or anti-Mouse IHC Kit (MaxVision) at 37 °C for 20 min. Then sections were incubated in blocking buffer from the same kit at 37 °C for 1 h followed by incubation with primary antibodies listed in the Table S3 at 4 °C overnight. Sections were then incubated with secondary antibodies from the same kits at 37 °C for 1 h. After staining with Diaminobenzidine (DAB), sections were counterstained with hematoxylin to visualize nuclei.

For TRAP staining, Acid Phosphatase Kit (Sigma-Aldrich) was applied to rehydrated paraffin-embedded sections according to the manufacturer’s instructions.

At last, sections were mounted with neutral mounting medium (Sinopharm). Images were obtained and displayed by section scanning using Scan digital pathology slides (3DHISTECH, Pannoramic 250 Flash III) and CaseViewer Software (3DHISTECH, 2.4.0).

### IF staining, TUNEL staining, and phalloidin staining

For tissue IF staining, frozen sections were blocked with 3% BSA at 37 °C for 1 h. Following incubation with primary antibodies overnight, sections were incubated with secondary antibodies listed in the Table S3 at 37 °C for 1 h.

For TUNEL staining, paraffin-embedded sections were stained with the One-step TUNEL In Situ Apoptosis Kit (Red, Elab Fluor 594; Elabscience) according to the manufacturer’s instructions.

For Phalloidin staining, frozen sections were stained with Rodamine-Phalloidin (Beyotime, 1:100) at room temperature for 1 h.

Finally, sections were mounted with DAPI-containing antifade mounting medium (ZSGB-Bio). Images were acquired using a DM4000B fluorescence microscope.

### ELISA assays

For ELISA assays, murine blood samples were collected from the posterior orbital venous plexus. After being set overnight, blood samples were centrifuged at 1 000 × *g* for 20 min, and the serum samples were collected. Concentrations of PINP and CTX-1 were detected using ELISA kits listed in the Table S3 according to manufacturers’ instructions.

### ARS staining, ORO staining and TUNEL staining

For ARS staining, BMSCs were cultured in the osteoblastic differentiation medium for 2 weeks, fixed with 4% PFA, and incubated with ARS for 5 min at room temperature. The cells were then gently washed with distilled water and dried. Images were acquired under consistent natural light conditions. For quantitative analysis, the ARS was solubilized using 10% cetylpyridinium chloride in sodium phosphate (NaP) buffer, and absorbance was measured at 540 nm.

For ORO staining, BMSCs were cultured in the adipocytic differentiation medium for 10 days, fixed with 4% PFA, and stained with 0.15% ORO (Solarbio). Positive cells containing lipid droplets were counted and analyzed.

For TUNEL staining, MLO-Y4 cells were seeded onto round coverslips (Nest), transfected with *Mdm2* siRNA and cultured for 4 days. Cells were then stained with One-step TUNEL In Situ Apoptosis Kit (Red, Elab Fluor 594; Elabscience).

### Flow cytometry analysis of cell lines

For flow cytometry analysis, at 4 days after transfection with *Mdm2* or scramble siRNA, MLO-Y4 cells were trypsinized, stained with Annexin V-FITC/PI Apoptosis Kit (Elabscience) according to the manufacturer’s instructions, and subjected to FACS Fortessa (BD Biosciences). Apoptosis was quantified as Annexin V-FITC positive cells with or without PI staining.

### In situ PLA

The interaction between MDM2 and TRPM8 was demonstrated using the Duolink proximity ligation assay kit (Sigma-Aldrich) as previously described.^63^ Briefly, MLO-Y4 cells were incubated with anti-MDM2 (mouse, 1:100) and anti-TRPM8 (rabbit, 1:50) antibodies overnight at 4 °C. Secondary antibodies conjugated to DuoLink PLA probes (Minus/Plus) were applied to the cells and incubated at 37 °C for 1 h. Positive signals were visualized as distinct green punctate fluorescence signals and captured by a Leica DM4000B microscope.

### Isolation and characterization of apovs

Apovs were harvested from the medium of MLO-Y4 cells at 4 days after transfection with *Mdm2* siRNA using sequential centrifugation as previously described.^38^ Briefly, cell debris was removed by sequential centrifugation including 800 × *g* for 10 min and 2 000 × *g* for 10 min at 4 °C. The supernatants were collected and centrifuged at 16 000 × *g* for 30 min at 4 °C to collect apovs. Apovs were also harvested from MLO-Y4 cells with First procaspase activating compound (PAC-1, 100 μmol/L, MedChemExpress) treatment for 8 h.

For NTA, MLO-Y4 cells-derived apovs were diluted in PBS and analyzed by ZetaView Particle Metrix (Particle Matrix, PMX-120) together with 100 nm PS beads (Thermo Fisher, 3100 A) for calibration. NTA software was used to measure the concentration of nanoparticles (particles/mL). The morphology of apovs was analyzed by Transmission electron microscopy (HITACHI, HT7700). The surface markers of apovs were examined by immunofluorescence and immunoblotting analysis.

### In vivo phagocytosis experiment

For in vivo tracking of apovs, MLO-Y4 cells-derived apovs were isolated, labeled with PKH26 (Solarbio), and suspended in 200 µL PBS at a concentration of 500 ng/µL. The suspended apovs were intravenously injected into mice at 3 weeks through the tail vein. Mice were sacrificed 24 h later and femurs were collected. BMSCs were identified by immunofluorescence of CD105. Fluorescent images were captured by Confocal microscope (Zeiss, LSM880).

### In vitro phagocytosis experiment

BMSCs were seeded onto coverslips at a density of 1 × 10⁴ cells/cm². MLO-Y4 cells-derived apovs were labeled with PKH26 (Solarbio) and added to the culture medium of BMSCs. After culturing for 0, 2, 6, and 24 h, BMSCs were fixed, permeabilized, and stained with 488-conjugated phalloidin (Beyotime) at room temperature for 1 h.

MLO-Y4 cells were transfected with *pFROG-Egfp*-*Trpm8* or *pCMV*-*Myc*-*Pirt*. After 48 h, cell apoptosis was induced by supplementing with 100 μmol/L PAC-1. After *pCMV*-*Myc*-*Pirt* transfected MLO-Y4 derived apovs were stained with 488 conjugated MYC tag, apovs with EGFP autofluorescence and apovs with MYC tag were added to PKH26-labeled BMSCs separately and cultured overnight.

Then the BMSCs on coverslips were fixed and mounted with DAPI-containing antifade mounting medium. Fluorescent images were captured by Confocal microscope (Zeiss, LSM880).

### Bulk RNA-seq and scRNA-seq analysis

Osteocytes were isolated from femurs and tibias as previously described and processed for bulk RNA-seq.^64,65^ Briefly, after removal of muscles and tendon tissues, bones were gently cut into small pieces and digested with 2.4 mg/mL Collagenase I (Biosharp) and 5 mmol/L EDTA to obtain osteocytes. Total RNA was extracted from osteocytes using Trizol reagent (Takara). Library construction and sequencing were carried out by Anoroad in Wuhan. After sample qualification, raw reads were filtered and aligned to the reference sequences. Gene expression levels were quantified for visualization using FPKM values, whereas differential expression analysis was performed using DESeq2 based on raw read count data. Genes in *Mdm2*^*cKO*^ compared with control mice with fold changes >2 and *q* < 0.05 were selected as significantly differentially expressed genes.

For scRNA-seq, bone marrow cells were isolated through flushing, while cells from bone pieces were digested with 2.4 mg/mL Collagenase I and 5 mmol/L EDTA as previously described.^66,67^ Specifically, periosteum cells were digested by Collagenase I for two consecutive 15 min from bone tissues and discarded. Osteoblasts and osteocytes were obtained by sequential digestion with Collagenase I for two consecutive 15 min, then EDTA for 20 min, then Collagenase I for 20 min, then EDTA for 20 min and finally Collagenase I for 20 min on a shaker with 90 r/min at 37 °C. Each sample contained cells from five 2-month-old male mice. Hematopoietic cell depletion was achieved using the Lineage Cell Depletion Kit (Miltenyi Biotec) according to the manufacturer’s instructions, followed by fluorescence-activated cell sorting to enrich CD45^-^ TER119^-^ CD71^-^ cells by flow cytometry. A single cell library was generated with Chromium Next GEM Single Cell 3’ Kit v3.1 (10x Genomics) and sequenced on an Illumina NovaSeq 6000 system, targeting >30 000 reads per cell for robust gene coverage. Raw sequencing data were filtered and processed with CellRange-7.0.0 (https://support.10xgenomics.com/single-cell-geneexpression/software/pipelines/latest/what-is-cell-ranger), which includes the following steps: demultiplexing by cell barcodes and UMIs, alignment of spliced reads to the reference genome, and UMI-based gene expression quantification for each gene and cell. Potential doublets were identified and removed using the scDblFinder package (v1.12.0) for each sample. Quality control was performed to filter low quality cells using the following criteria: 1. Cells with fewer than 200 detected genes were excluded; 2. Cells with extreme gene counts were filtered by removing cells in the top and bottom 2% quantiles; 3. Cells with total UMI counts in the top or bottom 2% were discarded; 4. Cells with mitochondrial gene expression exceeding 20% of total reads were eliminated; 5. Low-complexity cells displaying the novelty score of log_10_(Genes per UMI) > 0.8 were excluded to ensure high-quality transcriptional profiles. Raw count matrices were processed using Seurat (v4.3.0) in R (v4.2.3). For each sample, gene expression was normalized and variance stabilized via SCTransform, selecting 3 000 highly variable genes (HVGs) while excluding housekeeping genes, TCR/BCR, and immunoglobulin genes (IGV). Multiple samples were combined and batch-corrected using IntegrateData. Integrated data were centered and subjected to principal component analysis (PCA, 50 dimensions). Clustering was performed using FindNeighbors and FindClusters. Nonlinear dimensionality reduction was carried out via UMAP for visualization. Automated annotation was performed using SingleR, PanglaoDB and CellMarker. Manual refinement was applied based on canonical marker genes. FindAllMarkers function was applied in Seurat to detect DEGs among different clusters of the datasets. Pathway enrichment analysis was carried out by R package clusterProfiler (version 4.2.3).

Clusters of interest (e.g., clusters 0 and 5) were subsetted and re-analyzed: For re-normalization, samples were split and reprocessed with SCTransform; Batch correction was reapplied via IntegrateData; PCA (50 dimensions) was applied followed by FindNeighbors and FindClusters; Finally, UMAP and marker-based annotation were repeated.

### RT-qPCR and immunoblotting analysis

RNA isolation and RT-qPCR analysis were performed as previously described.^68^ The specific primers for gene expression analysis are listed in Table S3.

For cell protein extraction, cells were incubated in NP-40 lysis buffer (Beyotime) containing proteinase inhibitor cocktail (MedChemExpress) at 4 °C for 10 min. After centrifugation at 13 000 × *g* at 4 °C for 10 min, the supernatants were collected for the following experiments.

For femur protein extraction, femurs were collected, cut into small pieces on ice, and thoroughly ground using the tissue grinding low temperature homogenizer (TIANGEN, TGrinder H24R). Briefly, each sample was treated with 10 cycles at −10 °C (each cycle included grinding at 4 000 r/min for 60 s, then a pause for 10 s). After centrifugation at 12 000 × *g* for 30 min, supernatants were collected.

Immunoblotting analysis was performed as previously described.^16^ Blot membranes were incubated with primary antibodies listed in the Table S3 at 4 °C overnight. After incubation with HRP-conjugated secondary antibodies (Santa Cruz) at 37 °C for 1 h, blots were developed using an enhanced chemiluminescene (Millipore). Images were exposed in ChemiDoc XRS chemiluminescence imaging system (Bio-rad).

### Co-IP assay

Co-IP assay was performed as previously described.^16^ Briefly, cells were transfected with *pCMV*-*Flag*-*Mdm2*, *pFROG*-*Egfp*-*Trpm8*, and *pCMV-HA*-*Ub*. After 48 h, cell proteins were collected, incubated with primary antibodies overnight and then with 50 μL Protein A/G Magnetic Beads (Bimake) at room temperature for 1 h. Samples were next analyzed by immunoblotting.

### Dual luciferase reporter assay

HEK293T cells were cultured in 48-well plates and transfected with *pGL3*-*Trpm8*-promoter plasmids or *pGL3*-*Basic* vector together with *pRL*-*TK* Renilla luciferase expression vector (Progema) as well as MDM2 and p53 overexpression plasmids. Cells were lysed 48 h after the transfection. Luciferase activities were measured by Dual Luciferase Reporter Gene Assay Kit (Beyotime) and Dual Luciferase reporter assay system (Promega).

### Quantification and statistical analysis

All experiments were independently repeated at least three times, with biological replicate details listed in the figure legends. Data were presented as mean ± SD using GraphPad Prism 8. For comparisons between two groups, two-tailed unpaired Student’s *t*-tests were used. For comparisons among multiple groups, 1-way analysis of variance (ANOVA) was used. Details of the scRNA-seq statistical analysis are described in the Bulk RNA-seq and scRNA-seq Analysis section. *P* < 0.05 was considered statistically significant, and sample sizes are shown in the figure legends. Asterisks indicate significant differences with *P* < 0.05. Multiple asterisks denote lower *P*-values, as shown in the figure legends.

### Materials availability

This study did not generate new unique reagents.

## Supplementary information

Supplementary Information

Figure S1

Figure S2

Figure S3

Figure S4

Figure S5

Figure S6

Figure S7

Figure S8

Figure S9

Figure S10

Figure S11

## Data Availability

Data are available upon request to the lead contact. Sequencing data are available at the Gene Expression Omnibus (https://www.ncbi.nlm.nih.gov/geo/) under accession number GEO: GSE304502. Any additional information required to reanalyze the data reported in this work paper is available from the lead contact upon request.
