# Supplementary material for: Apoptosis of Mdm2-deficient osteocytes enhances osteogenesis through TRPM8-enriched apoptotic vesicles
Source: Bone Res. 2026 Aug 3;14:78. doi: 10.1038/s41413-026-00570-0 (PMC13429674; doi:10.1038/s41413-026-00570-0)
Supplement: Supplementary file 1 — Supplementary Information [file 41413_2026_570_MOESM1_ESM.docx]

Supplementary Materials for

**Apoptosis of *Mdm2*-deficient osteocytes enhances osteogenesis through TRPM8-enriched apoptotic vesicles**

Xin Liu, Huiwen Zheng, Aochen Wang, Jianing Liu, Zhi Chen, YiPing Chen, Guobin Yang, Di Chen, Yufeng Zhang^*^, Guohua Yuan*

*Correspondence:

Yufeng Zhang, [zyf@whu.edu.cn](mailto:zyf@whu.edu.cn).

Guohua Yuan, yuanguohua@whu.edu.cn

**Supplementary Figure Legends**

**Fig. S1 Inactivation of *Mdm2* in osteocytes leads to high bone mass phenotype in mice.**

**a** Immunofluorescence showing the expression of MDM2 in MLO-Y4 cells, an osteocyte cell line.

**b** The expression of MDM2 in the osteocytes of femurs from mice at 1 month old (1M) and 2M, and successful deletion of *Mdm2* in the osteocytes of *Mdm2* conditional knockout (*Mdm2*^cKO^) mice at 2M by immunohistochemistry.

**c** X-ray images showing the femurs in *Mdm2*^cKO^ mice compared with control littermates at 3M and 5M.

**d** Hematoxylin and eosin (HE) staining of sections of the femurs from *Mdm2*^cKO^ mice and control littermates at 1M, 2M, 3M and 5M. Histomorphometric quantification of Tb.N., Tb.Th. and Cort.Th. was presented (n=5 males).

**e** μ-CT images showing the third and the fifth lumbar vertebrae (L3 and L5) in *Mdm2*^cKO^ mice and control littermates at 3M.

**f** Three-dimensional reconstruction of μ-CT images in *Mdm2*^cKO^ mice and control littermates at 3M. Quantitative analysis of BV/TV, Tb.Th., Tb.N., and Tb.Sp. was presented (n=5 males).

**g** Femur lengths of *Mdm2*^cKO^ and control littermates at 1M and 2M. The average lengths were calculated. (n=5 males).

**h** Growth plate in the femurs of *Mdm2*^cKO^ and control littermates at 1M by HE staining. Histomorphometric quantification of the PZ height and HZ height was presented (n=5 males).

Data information: Scale bars: 20 μm (**a**); 50 μm (**b**); 10 μm (**b** (inserted magnifications); 200 μm (**d** (a, b, e, f, i, j, m, n)); 100 μm (**d** (c, d, g, h, k, l, o, p), **h**); 1 mm (**e**, **f**); 10 mm (**g**). Tb, trabecular bone; Cort, cortical bone; BM, bone marrow; DFP, distal femoral physis; BV/TV, bone volume over total volume; Tb.Th., trabecular thickness; Tb.N., trabecular number; Tb.Sp., trabecular separation; Cort.Th., cortical thickness; PZ, Proliferative layer zone; HZ, Hypertrophic layer zone. Data are presented as mean±SD (n=5/group); *P<0.05, **P<0.01; 2-tailed unpaired Student’s t test (**d**, **f**, **g**, **h**).

**Fig. S2 Regulation of osteocyte apoptosis by the MDM2-p53 axis.**

**a** GO analysis of the DEGs showing apoptosis related terms.

**b** The knockdown efficiencies of *Mdm2* and *Tp53* siRNAs in MLO-Y4 cells tested by RT-qPCR.

**c** Immunoblotting analysis of apoptotic markers BAX and CL-CAS3 in MLO-Y4 cells after transfection with indicated siRNAs. β-actin served as the loading control.

**d** Flow cytometry analysis using Annexin V and PI antibodies in MLO-Y4 cells transfected with indicated siRNAs. Quantification of the percentages of apoptotic cells percentage was presented.

**e** TUNEL staining and quantification of the TUNEL positive cells in MLO-Y4 cells transfected with indicated siRNAs.

Data information: Scr, Scramble siRNA; *Mdm2* Si, *Mdm2* siRNA; *Tp53* Si, *Tp53* siRNA. Scale bars: 50 μm (**e**). Data are presented as mean±SD (n=5/group (**d,** **e**); n=4/group (**b**)); **P<0.01; ANOVA (**b**, **d**, **e**).

**Fig. S3 Deletion of one allele of *Tp53* rescues the high bone mass phenotype of *Mdm2*^cKO^ mice.**

**a** p53 and p-p53 expression in the osteocytes of *Mdm2*^cKO^ mice compared with control littermates at 1M by immunofluorescence. Histomorphometric quantification of fluorescence intensity was presented (n=5 males).

**b** TUNEL staining and quantitative analysis showing apoptotic osteocytes in the femurs of *Mdm2*^cKO^, *Dmp1*-Cre; *Mdm2*^fl/fl^; *Tp53*^fl/+^ mice (*Mdm2*^cKO^; *Tp53*^fl/+^ mice), and control littermates at 2M and 5M (n=5 males). Dotted circles outlines empty lacunae. White arrows point to TUNEL positive cells.

**c** μ-CT images showing the femurs from *Mdm2*^cKO^, *Mdm2*^cKO^; *Tp53*^fl/+^ and control littermates at 2M.

**d** Three-dimensional reconstruction of μ-CT images in *Mdm2*^cKO^ and *Mdm2*^cKO^; *Tp53*^fl/+^ mice compared with control littermates at 2M. Quantitative analysis of BV/TV, Tb.Th., Tb.N., Tb.Sp. and Cort.Th. was presented (n=5 males).

**e** μ-CT images showing the femurs from *Mdm2*^cKO^, *Mdm2*^cKO^; *Tp53*^fl/+^ and control littermates at 5M.

**f** Three-dimensional reconstruction of μ-CT images in *Mdm2*^cKO^ and *Mdm2*^cKO^; *Tp53*^fl/+^ mice compared with control littermates at 5M. Quantitative analysis of BV/TV, Tb.Th., Tb.N., Tb.Sp., and Cort.Th. was presented (n=5 males).

Data information: BV/TV, bone volume over total volume; Tb.Th., trabecular thickness; Tb.N., trabecular number; Tb.Sp., trabecular separation; Cort.Th., cortical thickness. Scale bars: 10 μm (**a**); 50 μm (**b**); 500 μm (**c**, **d**, **e**, **f**). Data are presented as mean±SD (n=5/group); **P<0.01; 2-tailed unpaired Student’s t test (**a**); ANOVA (**b**, **d**, **f**).

**Fig. S4 *Mdm2*^cKO^ mice display increased bone resorption activity.**

**a** Immunoblotting analysis of osteoclast related proteins including NFATC1, MMP9 and TRAP in the femurs of *Mdm2*^cKO^ mice and control littermates at 1M.

**b** CTX-1 concentration in the serum of *Mdm2*^cKO^ mice compared with control littermates at 2M assessed by ELISA (n=4 males).

**c** TRAP staining in *Mdm2*^cKO^ mice compared with control littermates at 2M. Histomorphometric quantification of Oc.S/BS and Oc.N/BPm was presented (n=5 males).

**d**, **e** Immunohistochemistry of NFATC1 and CK as well as histomorphometric quantification of NFATC1 and CK positive cells in *Mdm2*^cKO^ mice compared with control littermates at 2M (n=5 males).

Data information: Oc.S/BS, osteoclast surface/bone surface; Oc.N/BPm, osteoclast number/bone perimeter; Tb, trabecular bone; BM, bone marrow. Scale bars: 100 μm (**c** (a, b), **d** (a, b), **e** (a, b)), 50 μm (**c** (c, d), **d** (c, d), **e** (c, d)). Data are presented as mean±SD (n=5/group (**c**, **d,** **e**); n=4/group (**b**)); **P<0.01; 2-tailed unpaired Student’s t test (**b**, **c**, **d**, **e**).

**Fig. S5 Periosteal bone formation is upregulated in *Mdm2*^cKO^ mice.**

**a** Double labeling of Calcein and Alizarin red S (ARS) in the periosteum of *Mdm2*^cKO^ mice and control littermates. The mineral apposition rate (MAR) was calculated.

**b, c** Immunohistochemistry of OCN and OSX as well as histomorphometric quantification of the positive cells in *Mdm2*^cKO^ and control littermates at 2M.

Data information: Cort, cortical bone; BM, bone marrow. Scale bars: 100 μm (**a** (a, b)), 50 μm (**a** (c, d), **b**, **c**). Data are presented as mean±SD (n=5/group); **P<0.01; 2-tailed unpaired Student’s t test (**a**, **b**, **c**).

**Fig. S6 Single-cell RNA-sequencing (scRNA-seq) of bone cells from *Mdm2*^cKO^ mice and control littermates.**

**a** Diagrammatic sketch showing bone sample preparation for scRNA-seq.

**b** Violin plots showing the expression levels of the marker genes representing 28 cell clusters.

**c** UMAP plots showing the expression of marker genes including *Postn*, *Dmp1*, *Cxcl12* and *Kitl* in cell clusters.

**d** Cluster 0 and 5 representing CAR were sorted out in the UMAP plot.

**e** Dot plots showing the expression levels of representative marker genes in each subcluster identified in Figure 2g. CAR, *Cxcl12* abundant reticular cells.

**Fig. S7 *Mdm2* deletion is necessary for apoptotic MLO-Y4 cells to promote osteogenesis in BMSCs.**

**a** Diagrammatic sketch of the co-culture strategy.

**b** Immunofluorescence and immunoblotting analysis of RUNX2 and OSX in BMSCs after co-culture in ODM with MLO-Y4 cells treated with scramble siRNA, PAC-1, or *Mdm2* siRNA. Quantification of RUNX2 and OSX positive cells was presented.

**c** ARS staining of BMSCs after co-culture in the ODM with MLO-Y4 cells treated with scramble siRNA, PAC-1, or *Mdm2* siRNA. Quantification of the eluted ARS dye was presented.

**d** Fluo-4AM staining in BMSCs after co-culture with MLO-Y4 cells treated with scramble siRNA, PAC-1, or *Mdm2* siRNA. Quantification of Fluo-4AM fluorescence intensity was presented.

Data information: MLO-Y4 cells were transfected with *Mdm2* (designated as Y4- *Mdm2* Si cells), scramble siRNA (designated as Y4-Scr cells), or treated with PAC-1 after being transfected with scramble siRNA (designated as Y4-PAC-1 cells). Scr, scramble siRNA; *Mdm2* Si, *Mdm2* siRNA; ODM, osteoblastic differentiation medium. Scare bars: 100 μm (**c**), 20 μm (**b**), 10 μm (**d**). Data are presented as mean±SD (n=5/group); **P<0.01; ANOVA (**b**, **c**, **d**).

**Fig. S8 Apovs derived from *Mdm2*-deficient osteocytes suppressed the adipocytic differentiation of BMSCs.**

**a** RT-qPCR showing the mRNA levels of *Pparγ* and *C/ebpα* in the BMSCs after co-culture with indicated MLO-Y4 cells.

**b** ORO staining showing the adipocytic differentiation of BMSCs after co-culture with indicated MLO-Y4 cells. Quantification of ORO positive areas was presented.

**c** RT-qPCR showing the mRNA levels of *Pparγ* and *C/ebpα* in the BMSCs treated with apovs derived from *Mdm2* knockdown MLO-Y4 cells or a solvent control under adipogenic induction.

**d** ORO staining of the BMSCs treated with apovs derived from *Mdm2* knockdown MLO-Y4 cells or a solvent control under adipogenic induction. Quantification of ORO positive areas was presented.

Data information: BMSCs were next co-cultured with the above MLO-Y4 cells in adipogenic induction medium for 10 days. ORO, Oil red O; Scr, Scramble siRNA; *Mdm2* Si, *Mdm2* siRNA; Y4*^Mdm2^* ^Si^-apovs, apovs derived from MLO-Y4 cells with *Mdm2* knockdown. Scale bars: 100 μm (**b**, **d**). Data are presented as mean±SD (n=5/group (**b**, **d**); n=4/group (**a**, **c**)); **P<0.01; 2-tailed unpaired Student’s t test (**a**, **b**, **c**, **d**).

**Fig. S9 TRPM8 and PIRT are specifically enriched in apovs derived from *Mdm2* knockdown MLO-Y4 cells.**

**a** Immunoblotting analysis of TRPM8 and PIRT in MLO-Y4 cells with or without *Mdm2* knockdown as well as in the apovs from MLO-Y4 cells treated with PAC-1 or *Mdm2* siRNA.

**b** Immunoblotting analysis of TRPM8 and PIRT in BMSCs after culture with apovs derived from MLO-Y4 cells treated with scramble siRNA, PAC-1, or *Mdm2* siRNA.

Data information: Y4*^Mdm2^* ^Si^-apovs, apovs derived from MLO-Y4 cells with *Mdm2* knockdown; Y4^PAC-1^-apovs, apovs derived from MLO-Y4 cells treated with PAC-1.

**Fig. S10 MDM2 negatively regulates TRPM8 post-translationally and transcriptionally.**

**a** In situ proximity ligation assay (PLA) using MDM2 and TRPM8 antibodies demonstrating the interaction between MDM2 and TRPM8 in MLO-Y4 cells. White arrows point to the PLA positive signals.

**b** Co-immunoprecipitation (co-IP) assays showing the physical interaction between the overexpressed FLAG-MDM2 and EGFP-TRPM8 in MLO-Y4 cells.

**c** Co-IP assays showing the ubiquitination of TRPM8 by overexpressed FLAG-MDM2 in HEK293T cells.

**d** CHX assay demonstrating that overexpressed MDM2 decreases the half-life of TRPM8 protein in MLO-Y4 cells. The cells were treated with CHX for 0, 4 and 8 hours. The relative levels of TRPM8 at different time points were measured.

**e** Immunoblotting analysis showing that MG132 treatment inhibited the degradation of TRPM8 but not Chloroquine treatment. Each inhibitor was added into the medium 4 hours before harvest.

**f** Dual luciferase assays in HEK293T cells after transfection with indicated plasmids.

**g** The mRNA level of *Trpm8* in MLO-Y4 cells transfected with indicated siRNAs assessed by RT-qPCR. Scale bars: 20 μm (**a**). Data are presented as mean±SD (n=4/group (**g**); n=3/group (**f**)); **P<0.01; ANOVA (**f**, **g**).

**Fig. S11 Intracellularly expressed MDM2 is required for the osteogenic activity.**

**a**, **b** EGFP signals in *Dmp1*-*Cre*; *R26R*^mTmG^ mice compared with mTmG control at 1M under the fluorescence microscope.

**c** Immunohistochemistry showing successful deletion of *Mdm2* in the osteoblasts of *Mdm2*^cKO^ mice compared with control littermates at 2M.

**d** Immunoblotting analysis of OSX and ALP in MC3T3-E1 cells after culture in the ODM for 4 days following transfection with *Mdm2* or scramble siRNA.

**e** Representative images showing ARS staining of MC3T3-E1 cells after culture in ODM for 2 weeks following transfection with *Mdm2* or scramble siRNA. Quantification of the eluted ARS dye was presented.

Data information: Ob, osteoblasts; Ocy, osteocytes; Tb, trabecular bone; Cort, cortical bone; BM, bone marrow. Scr, Scramble siRNA; *Mdm2* Si, *Mdm2* siRNA; ODM, osteoblastic differentiation medium. Scale bars: 200 μm (**a**), 100 μm (**b** (a, b, e, f), **e**), 50 μm (**c**), 10 μm (**b** (c, d, g, h), **c** (inserted magnifications)). Data are presented as mean±SD (n=5/group); **P<0.01; 2-tailed unpaired Student’s t test (**e**).

**Supplementary Tables**

**Table S1 Genotyping sequences used in this study.**

| **Genotype** | **Primers** | **Sequences** |
| --- | --- | --- |
| *Dmp1*-Cre | Forward | TGGAAGCTGACAGTAGGAAAC |
|  | Reverse 1 | TGACATCATCCCACGTACTTAAGC |
|  | Reverse 2 | TGGTGCACAGTCAGCAGGTTG |
| *Mdm2*^flox/flox^ | Forward | GGTCTTCCCATTTATGTATGT |
|  | Reverse | AAGAGTCTGTATCGCTTTCT |
| *Tp53*^flox/flox^ | Forward | GGTTAAACCCAGCTTGACCA |
|  | Reverse | GGAGGCAGAGACAGTTGGAG |
| *R26R*^mTmG^ | Forward | CTCTGCTGCCTCCTGGCTTCT |
|  | Reverse 1 | CGAGGCGGATCACAAGCAATA |
|  | Reverse 2 | TCAATGGGCGGGGGTCGTT |

**Table S2 siRNA sequences used in this study.**

| **siRNA** | **Forward (5'-3')** | **Reverse (3'-5')** |
| --- | --- | --- |
| Scramble | UUCUCCGAACGUGUCACGUTT | ACGUGACACGUUCGGAGAATT |
| *Mdm2* siRNA | GCUUCUCCCUGAAUGCCAUTT | AUGGCAUUCAGGGAGAAGCTT |
| *Tp53* siRNA | CCACUUGAUGGAGAGUAUUTT | AAUACUCUCCAUCAAGUGGTT |
| *Trpm8* siRNA | GCACCAGUCAAGAUUCCAATT | UUGGAAUCUUGACUGGUGCTT |

**Table S3 siRNA sequences used in this study.**

| **READENT or RESOURCE** | **SOURCE** | **IDENTIFIER** |
| --- | --- | --- |
| **Antibodies** |  |  |
| anti-Mdm2 | Abcam | Cat# ab16895, RRID:AB_2143534 |
| anti-p53 | Proteintech | Cat# 10442-1-AP, RRID:AB_2206609 |
| anti-p-p53 (Ser15) | Abclonal | Cat# AP0083, RRID:AB_2771380 |
| anti-Cleaved Caspase 3 | Abclonal | Cat# A11021, RRID:AB_2758369 |
| anti-Bax | Abclonal | Cat# A19684, RRID:AB_2862733 |
| anti-Runx2 | Boster | Cat# BM4700, RRID:AB_3081995 |
| anti-Osx | Abcam | Cat# ab209484, RRID:AB_2892207 |
| anti-ColI | Proteintech | Cat# 14695-1-AP, RRID:AB_2082037 |
| anti-Ocn | Cell Signaling Technology | Cat# 59757, RRID:AB_3246428 |
| anti-Trpm8 | Boster | Cat# BM5383, RRID:AB_2315502 |
| anti-Pirt | Proteintech | Cat# 20990-1-AP, RRID:AB_2878785 |
| anti-Flag | Abclonal | Cat# AE005, RRID:AB_2770401 |
| anti-Myc | Abclonal | Cat# AE010, RRID:AB_2770408 |
| anti-HA | Abclonal | Cat# AE008, RRID:AB_2770404 |
| anti-GFP | Abclonal | Cat# AE011, RRID:AB_2771922 |
| anti-CD105 | R&D Systems | Cat# AF1320, RRID:AB_354735 |
| anti-Nfatc1 | Zenbio | Cat# 251865, RRID:AB_10637567 |
| anti-Mmp9 | Abcam | Cat# ab283575, RRID:AB_2928971 |
| anti-Trap | Zenbio | Cat# R382344, RRID:AB_3692825 |
| anti-CK | Zenbio | Cat# R381730, RRID:AB_3672489 |
| anti-Alp | Abcam | Cat# ab229126, RRID:AB_3662672 |
| anti-CD9 | Abmart | Cat# T55337, RRID:AB_2747365 |
| anti-CD45 | BD Pharmingen | Cat# 557659, RRID:AB_396774 |
| anti-CD71 | BD Pharmingen | Cat# 553267, RRID:AB_394744 |
| anti-TER119 | BD Pharmingen | Cat# 557915, RRID:AB_396936 |
| anti-β-actin  anti-c-FOS  anti-c-JUN | Abclonal  Abcam  Abcam | Cat# AC028, RRID:AB_2769861  Cat# ab190289, RRID:AB_2893049  Cat# ab31419, RRID:AB_731604 |
| Alexa Fluor®594 Donkey anti Rabbit IgG（H+L） | Antgene | Cat# ANT030, RRID:AB_3107110 |
| Alexa Fluor®594 Donkey anti Mouse IgG（H+L） | Antgene | Cat# ANT029, RRID:AB_2813898 |
| Alexa Fluor®488 Donkey anti Rabbit IgG（H+L） | Antgene | Cat# ANT024, RRID:AB_3107111 |
| Alexa Fluor®488 Donkey anti Mouse IgG（H+L） | Antgene | Cat# ANT023, RRID:AB_2756710 |
| Alexa Fluor®488 Donkey anti Goat IgG（H+L） | Antgene | Cat# ANT025, RRID:AB_2866497 |
| HRP-Goat Anti-mouse IgG (H+L) | Antgene | Cat# ANT019, RRID:AB_3101880 |
| HRP-Goat Anti-rabbit IgG (H+L) | Antgene | Cat# ANT020, RRID:AB_3572247 |
| EasyBlot anti Rabbit IgG (HRP) | Genetex | Cat# GTX221666-01, RRID:AB_10620421 |
| EasyBlot anti Mouse IgG (HRP) | Genetex | Cat# GTX221667-01, RRID:AB_10728926 |
| **Chemicals and Reagents** |  |  |
| Collagenase I | Biosharp | BS163 |
| EDTA | HUSHI | H-10009617 |
| PAC-1 | MedchemExpress | HY-13523 |
| Calcein | Sigma-Aldrich | C0875 |
| Alizarin Red | Sigma-Aldrich | A5533 |
| Cetylpyridinium Chloride | Sigma-Aldrich | C0732 |
| Oil Red O | Solarbio | G1260 |
| Fluo-4AM | Beyotime | S1060 |
| MG132 | MedchemExpress | HY-13259 |
| Chloroquine | MedchemExpress | HY-17589A |
| RIPA | Beyotime | P0013B |
| PMSF | Beyotime | ST506 |
| NP-40 | Beyotime | P0013F |
| Protein A/G Beads | Selleck | B23202 |
| Cocktail | MedchemExpress | HY-K0010 |
| SDS-PAGE Sample Loading Buffer,5X | Biosharp | BL502A |
| Multicolor Prestained Protein Ladder | Epizyme | WJ103 |
| Primary Antibody Dilution Buffer | Beyotime | P0023A |
| Rabbit IgG | Beyotime | A7016 |
| Mouse IgG | Beyotime | A7028 |
| RNAiso Plus | TAKARA | 9108Q |
| Chloroform | Sigma-Aldrich | CX1060 |
| Isopropanol | Sinopharm | 10004160 |
| Cycloheximide | MedchemExpress | HY-12320 |
| Phosphotungstic Acid | Solarbio | G1870 |
| Actin-Tracker Green-488 | Beyotime | C2201S |
| Actin-Tracker Red-Rhodamine | Beyotime | C2207S |
| α-Minimum Essential Medium | Hyclone | SH30265.01 |
| Dulbecco's Modified Eagle Medium | Hyclone | SH30249.01 |
| Fetal Bovine Serum, Premium Plus | Gibco | A5669701 |
| Phosphate-Buffered Saline | Hyclone | SH30526.01 |
| Penicillin-Streptomycin | Hyclone | SV300010 |
| Trypsin (0.25%) | Hyclone | SH30042.01 |
| Sodium β-glycerophosphate | Sigma-Aldrich | G9422 |
| Ascorbic Acid | Sigma-Aldrich | 1043003 |
| Dexamethasone | Sigma-Aldrich | D4902 |
| Isobutylmethylxanthine | Sigma-Aldrich | I5879 |
| Indomethacin | Sigma-Aldrich | I7378 |
| Insulin | Sigma-Aldrich | I2643 |
| OCT | Sakura | 4583 |
| Hematoxylin Solution | Servicebio | G1004 |
| Eosin Solution | Servicebio | G1001 |
| Absolute Ethanol | Sinopharm | 10009218 |
| Sodium Hydroxide | HUSHI | 10019762 |
| n-Butanol | Sinopharm | 10002160 |
| Xylene | Sinopharm | 10023418 |
| Pepsin | ZSGB-Bio | ZLI-9014 |
| Neutral Mounting Medium | Sinopharm | 10004160 |
| Tween-20 | Servicebio | GC204002 |
| PBS | Servicebio | G0002 |
| TBS | Servicebio | G1001 |
| Triton X-100 | BioFroxx | 4240-100 |
| Bovine serum albumin, BSA | Beyotime | ST023 |
| 4',6-Diamidino-2-Phenylindole (DAPI) | ZSGB-Bio | ZLI-9557 |
| Glycine sodium salt | BioFroxx | 1415-100 |
| Tris base | BioFroxx | 1414-100 |
| Sodium Dodecyl Sulfate | BioFroxx | 1425-100 |
| Methanol | Sinopharm | 10014118 |
| Paraformaldehyde (4%) | Servicebio | G1101 |
| Glutaraldehyde (25%) | Sinopharm | 30092436 |
| Bismuth Acetate (1%) | Sinopharm | 30106920 |
| Hieff® qPCR SYBR Green Master Mix(Low Rox Plus) | Yeasen | 11202ES03 |
| Molecular Biology Grade Water | Hyclone | SH30538.02 |
| Lipofectamine 3000 | Thermo | L3000015 |
| Opti-MEM™ I Reduced-Serum Medium | Thermo | 31985070 |
| 1M Tris-HCl (pH=8.0) | Beyotime | ST780 |
| 50 X TAE | Beyotime | ST716 |
| Agarose | Sangon | A620014-0100 |
| 100 bp DNA ladder | Yeasen | 10507ES60 |
| Protein Marker | Epizyme | WJ102 |
| GelRed | Yeasen | 10202ES76 |
| 2 × Hieff® HotStart PCR Genotyping Master Mix | Yeason | 10108ES03 |
| PKH26 | Solarbio | D0030 |
| Phosphotungstic acid (2%) | Solarbio | G1870 |
| **Reagent Test Kits** |  |  |
| One-step TUNEL In Situ Apoptosis Kit | Elabscience | E-CK-A322 |
| Annexin V-FITC/PI Apoptosis Kit | Elabscience | E-CK-A211 |
| Lineage Cell Depletion Kit, mouse | Miltenyi Biotec | 130-090-858 |
| PINP ELISA | Ruixin Biotech | RX203163M |
| CTX-1 ELISA | Ruixin Biotech | RX203324M |
| Enhanced BCA Protein Assay Kit | Beyotime | P0010 |
| Dual Luciferase Assay | Beyotime | RG027 |
| TRAP Staining Kit | Sigma-Aldrich | 387A |
| DAB Color Development Kit | ZSGB-Bio | ZLI-9017 |
| HRP Polymer anti-Rabbit IHC Kit | Maxvision | KIT-5004 |
| HRP Polymer anti-mouse IHC Kit | Maxvision | KIT-5001 |
| WesternBright ECL kit | Advansta | K-12045-D20 |
| ABScript HII Reverse Transcriptase | Abclonal | RK26507 |
| Duolink® In Situ PLA® Probe Anti-Mouse PLUS | Sigma-Aldrich | DUO92001 |
| Duolink® In Situ PLA® Probe Anti-Rabbit MINUS | Sigma-Aldrich | DUO92005 |
| Duolink® In Situ Detection Reagents Green | Sigma-Aldrich | DUO92014 |
| Duolink® In Situ Wash Buffers, Fluorescence | Sigma-Aldrich | DUO82049 |
| **Software and algorithms** |  |  |
| CTAn v.1.18 | Bruker | <https://blue-scientific.com/news/2018/07/bruker-ctan-micro-ct-software/> |
| Mimics Medical 20.0 | Materialise | <https://www.materialise.com/en/healthcare/mimics/mimics-core> |
| 3-Matics Medical 20.0 | Materialise | <https://www.materialise.com/en/healthcare/mimics/3-matic-medical> |
| Graphpad Prism 10.1.2 | Graphpad Software | N/A |
| CaseViewer 2.2 | 3DHISTECH | N/A |
| Image Studio 6.0 | LICORbio | <https://www.licorbio.com/image-studio> |
| Cytexpert 2.6.0.105 | Beckman | <https://www.beckman.com/flow-cytometry/research-flow-cytometers/cytoflex/software> |
| Image J | National Institutes of Health | [https://imagej.net/ij/.](https://imagej.net/ij/) |
| R Version 4.2.3 | R Foundation | <https://cran.r-project.org/bin/windows/base/old/4.2.3/R-4.2.3-win.exe> |
| Cellranger-7.0.0 | 10x Genomics | <https://support.10xgenomics.com/single-cell-geneexpression/software/pipelines/latest/what-is-cell-ranger> |

**Table S4 Primer sequences used in this study for RT-qPCR.**

| **Gene** | **Forward (5'-3')** | **Reverse (3'-5')** |
| --- | --- | --- |
| *Gapdh* | TGTGTCCGTCGTGGATCTGA | TTGCTGTTGAAGTCGCAGGAG |
| *Mdm2* | CCAACCATCGACTTCCAGCAGCATT | GATTGGCTGTCTGCACACTGGG |
| *Tp53* | ATGGAGGAGCCGCAGTCAGATC | CCATTGTTCAATATCGTCCGGG |
| *Pparγ* | GATGGAAGACCACTCGCATT | GGATCCGGCAGTTAAGATCA |
| *C/ebpα* | CAAGAACAGCAACGAGTACCG | GTCACTGGTCAACTCCAGCAC |
| *Trpm8* | CCAAGGAGTTTCCAACAGACG | CGTGGCTTCAAAGCAAAGTTT |
| *Pirt*  *c-Fos*  *c-Jun* | TAGACGAGAGGTCTCCAGAGT  AGAGCGGGAATGGTGAAG  CGCACAGCCCAGGCTAAC | CCAGTTGCTTTTGGGTGTGG  GGATTCTCCGTTTCTCTTCC  TGAGGGCATCGTCGTAGAA |
