# Supplementary figures and images for: Apoptosis of Mdm2-deficient osteocytes enhances osteogenesis through TRPM8-enriched apoptotic vesicles
Source: Bone Res. 2026 Aug 3;14:78. doi: 10.1038/s41413-026-00570-0 (PMC13429674; doi:10.1038/s41413-026-00570-0)

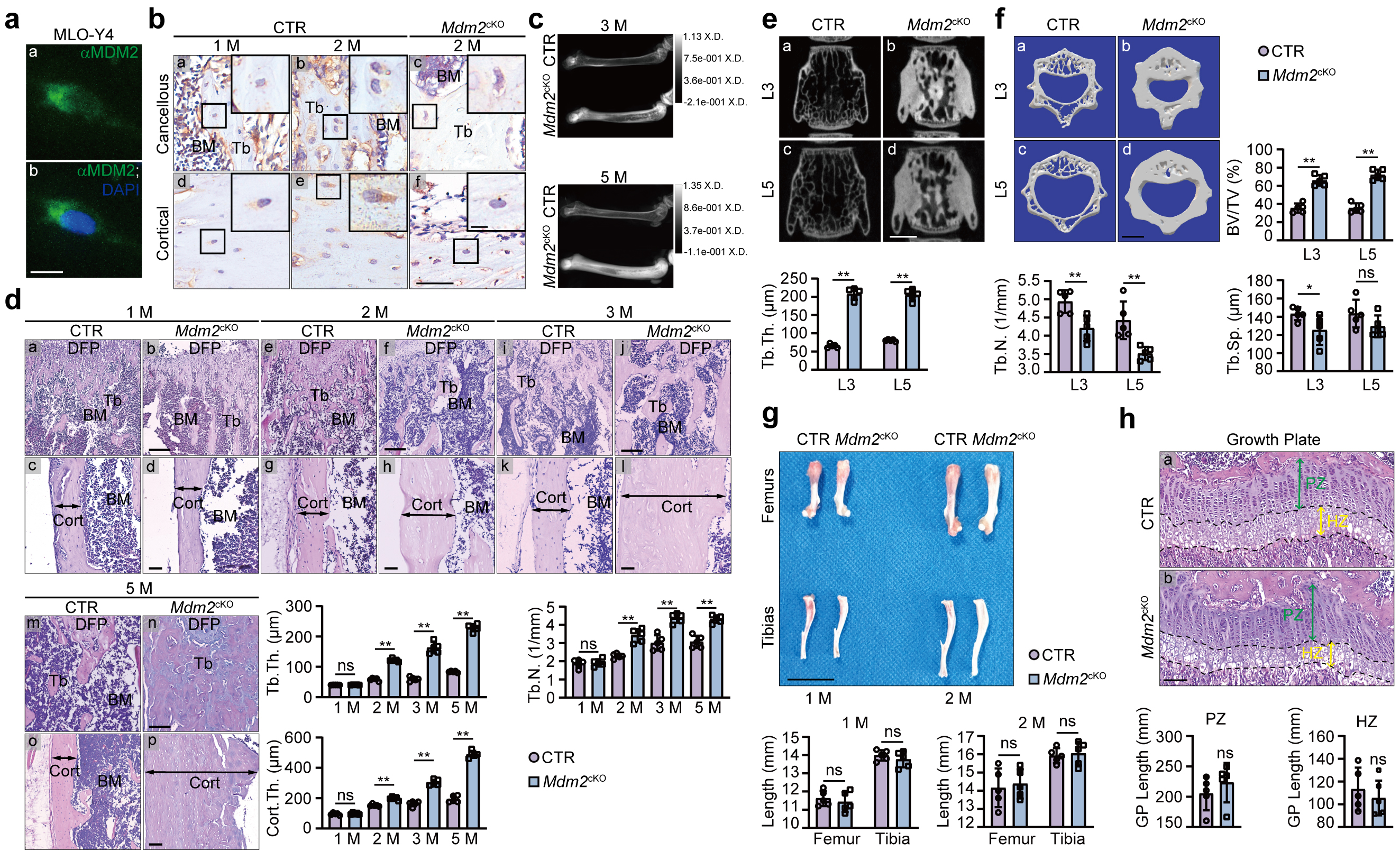

Supplement: Supplementary file 2 — Figure S1 [file 41413_2026_570_MOESM2_ESM.jpg]

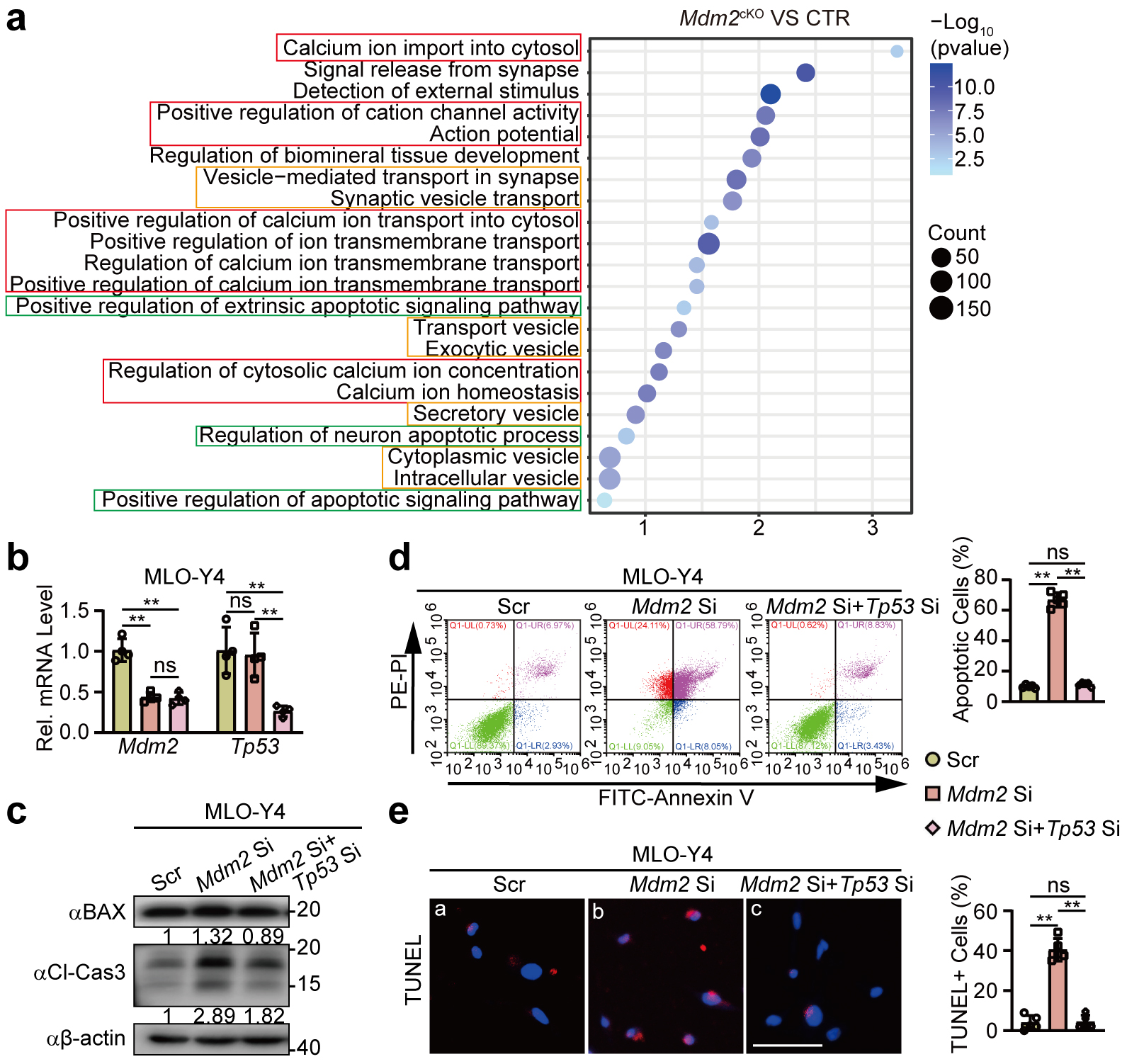

Supplement: Supplementary file 3 — Figure S2 [file 41413_2026_570_MOESM3_ESM.jpg]

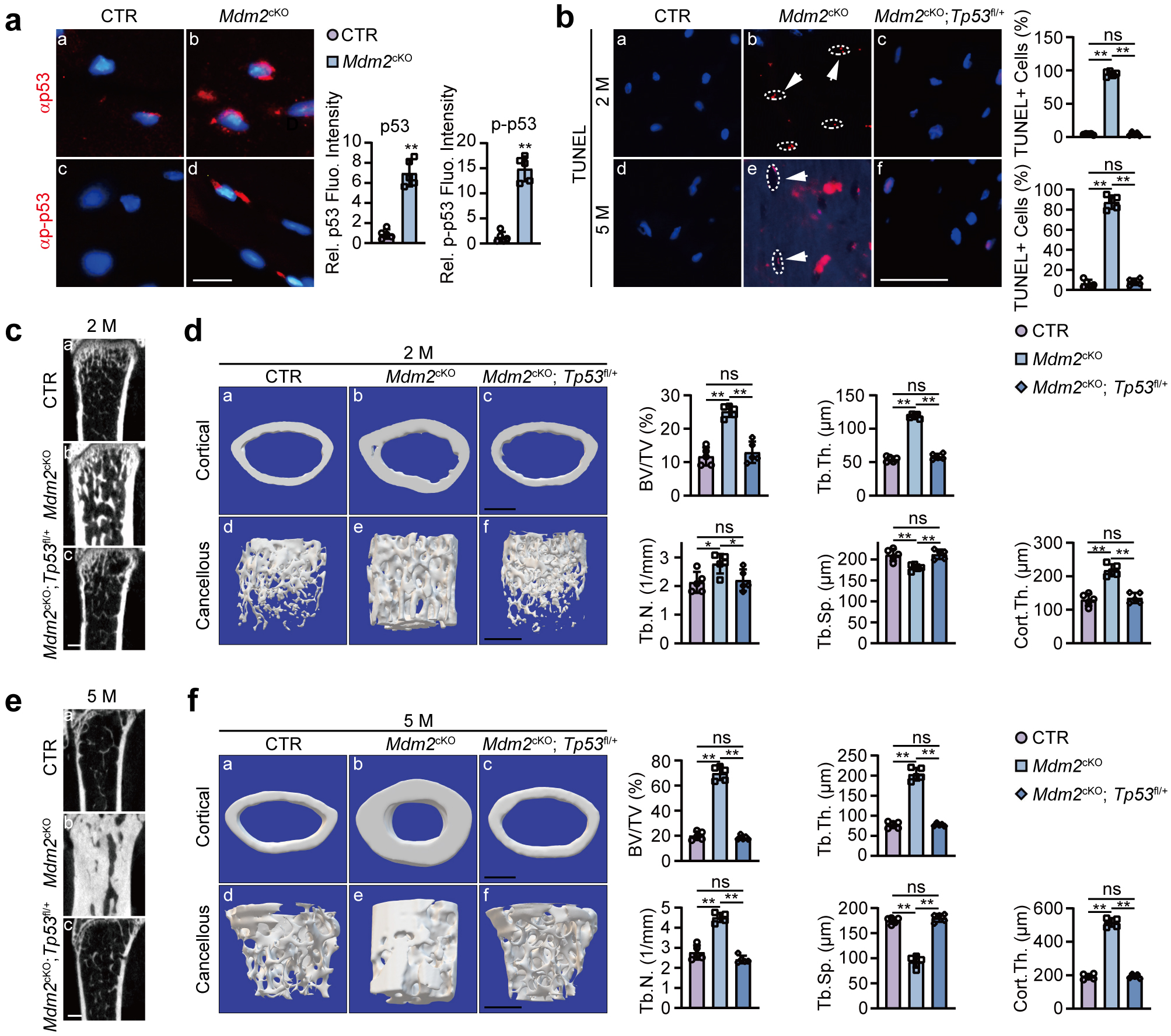

Supplement: Supplementary file 4 — Figure S3 [file 41413_2026_570_MOESM4_ESM.jpg]

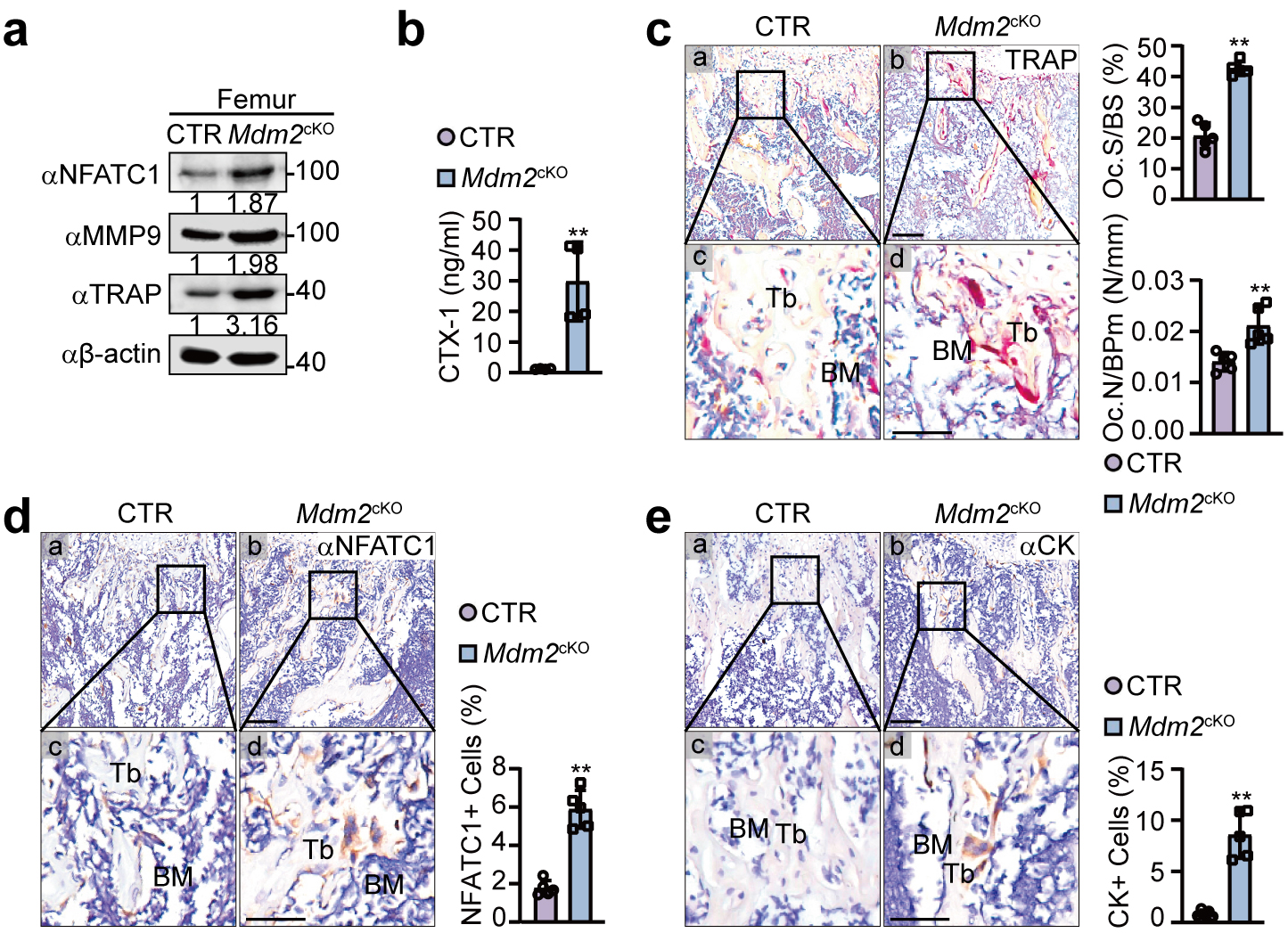

Supplement: Supplementary file 5 — Figure S4 [file 41413_2026_570_MOESM5_ESM.jpg]

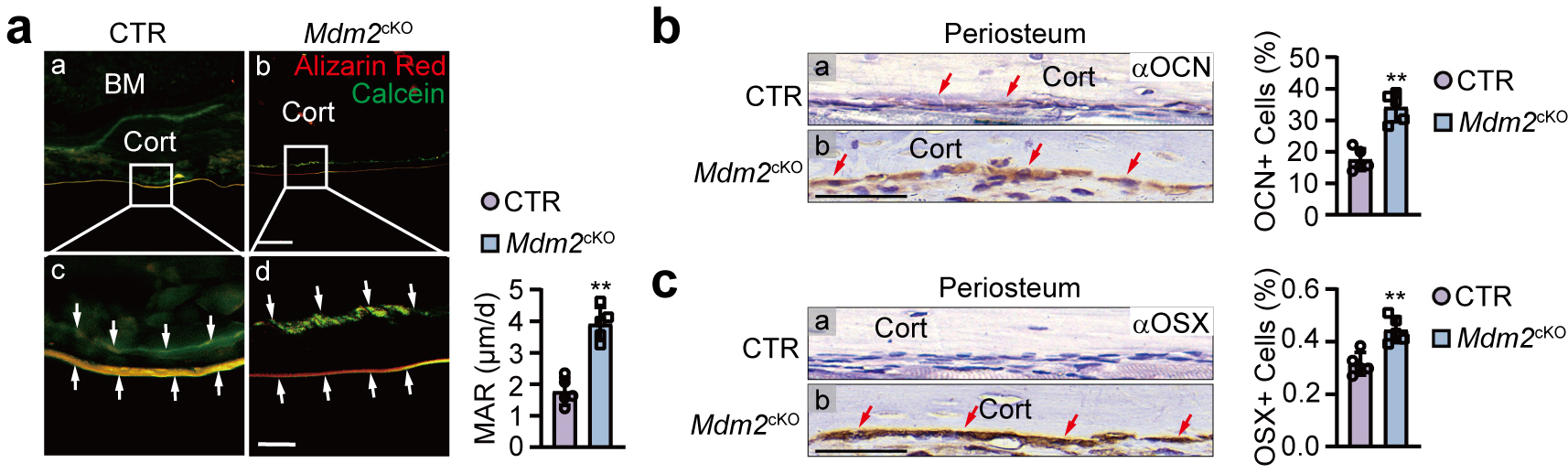

Supplement: Supplementary file 6 — Figure S5 [file 41413_2026_570_MOESM6_ESM.jpg]

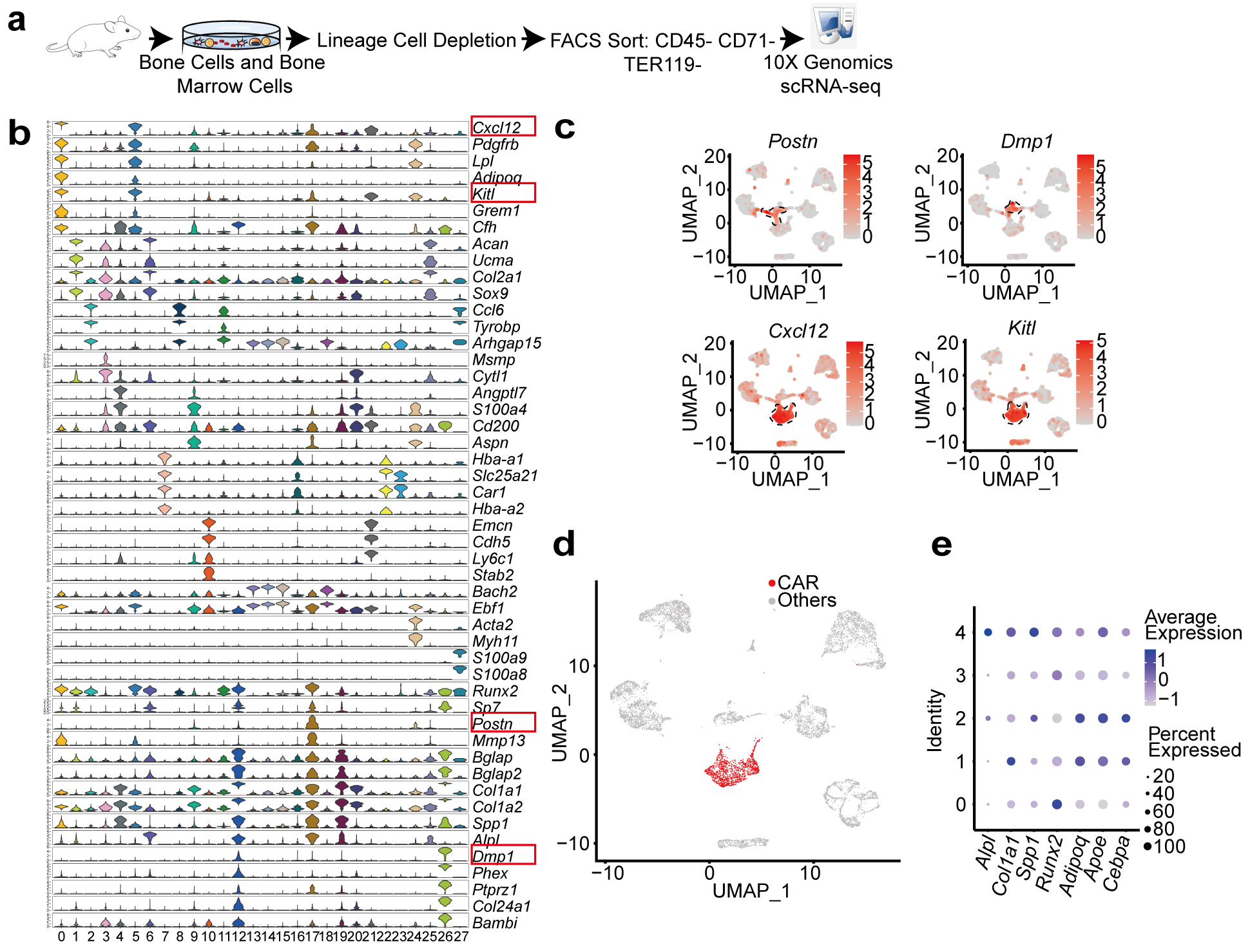

Supplement: Supplementary file 7 — Figure S6 [file 41413_2026_570_MOESM7_ESM.jpg]

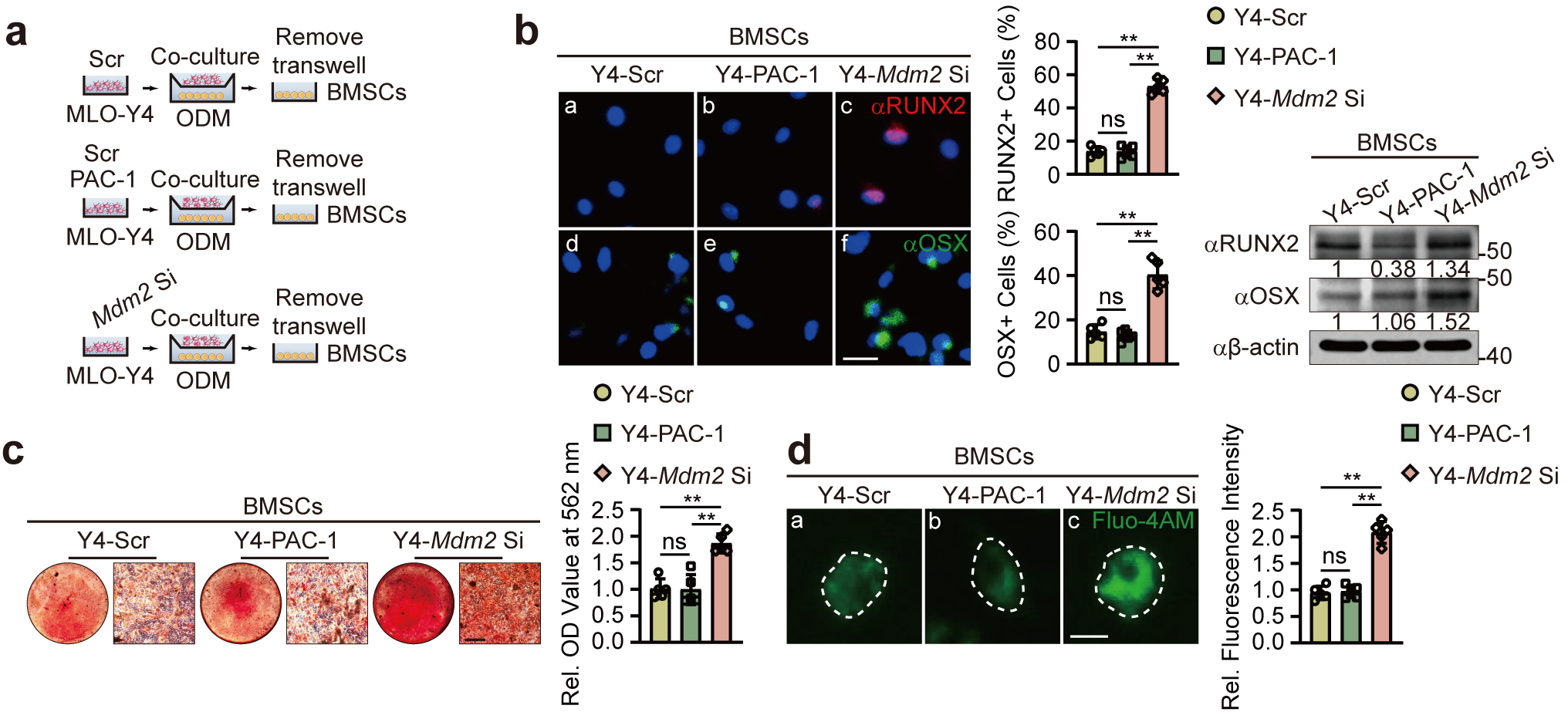

Supplement: Supplementary file 8 — Figure S7 [file 41413_2026_570_MOESM8_ESM.jpg]

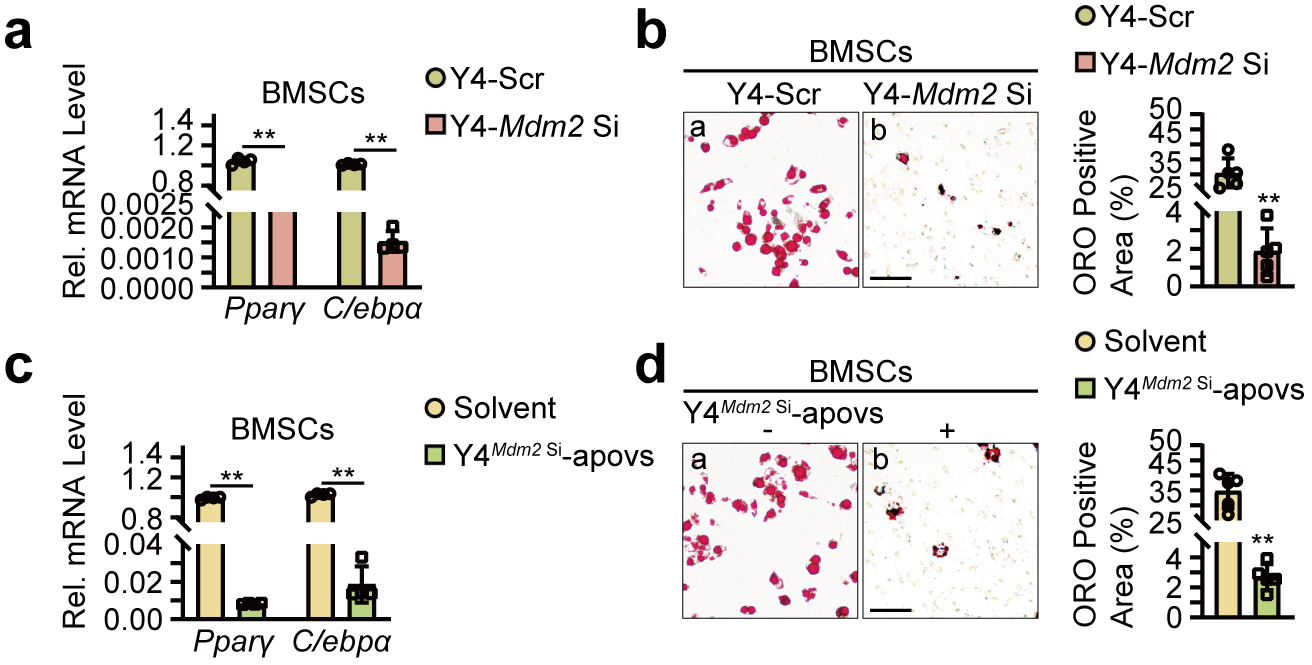

Supplement: Supplementary file 9 — Figure S8 [file 41413_2026_570_MOESM9_ESM.jpg]

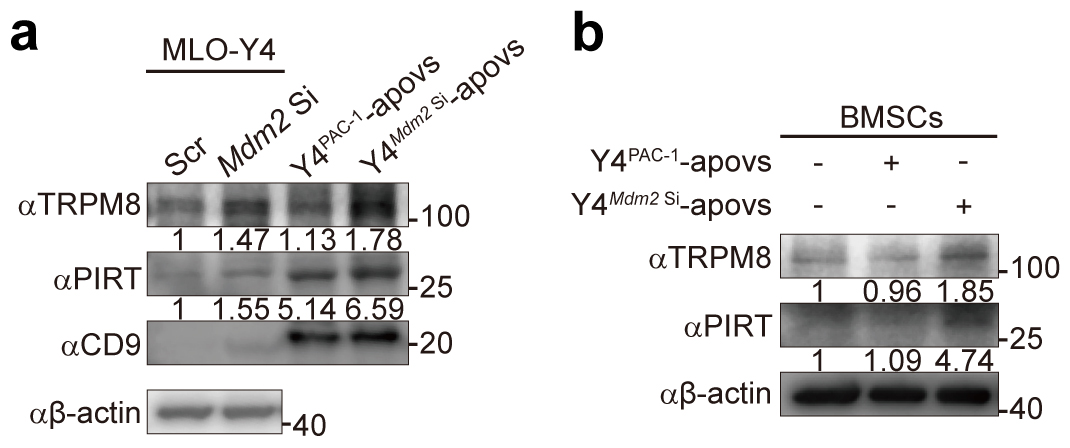

Supplement: Supplementary file 10 — Figure S9 [file 41413_2026_570_MOESM10_ESM.jpg]

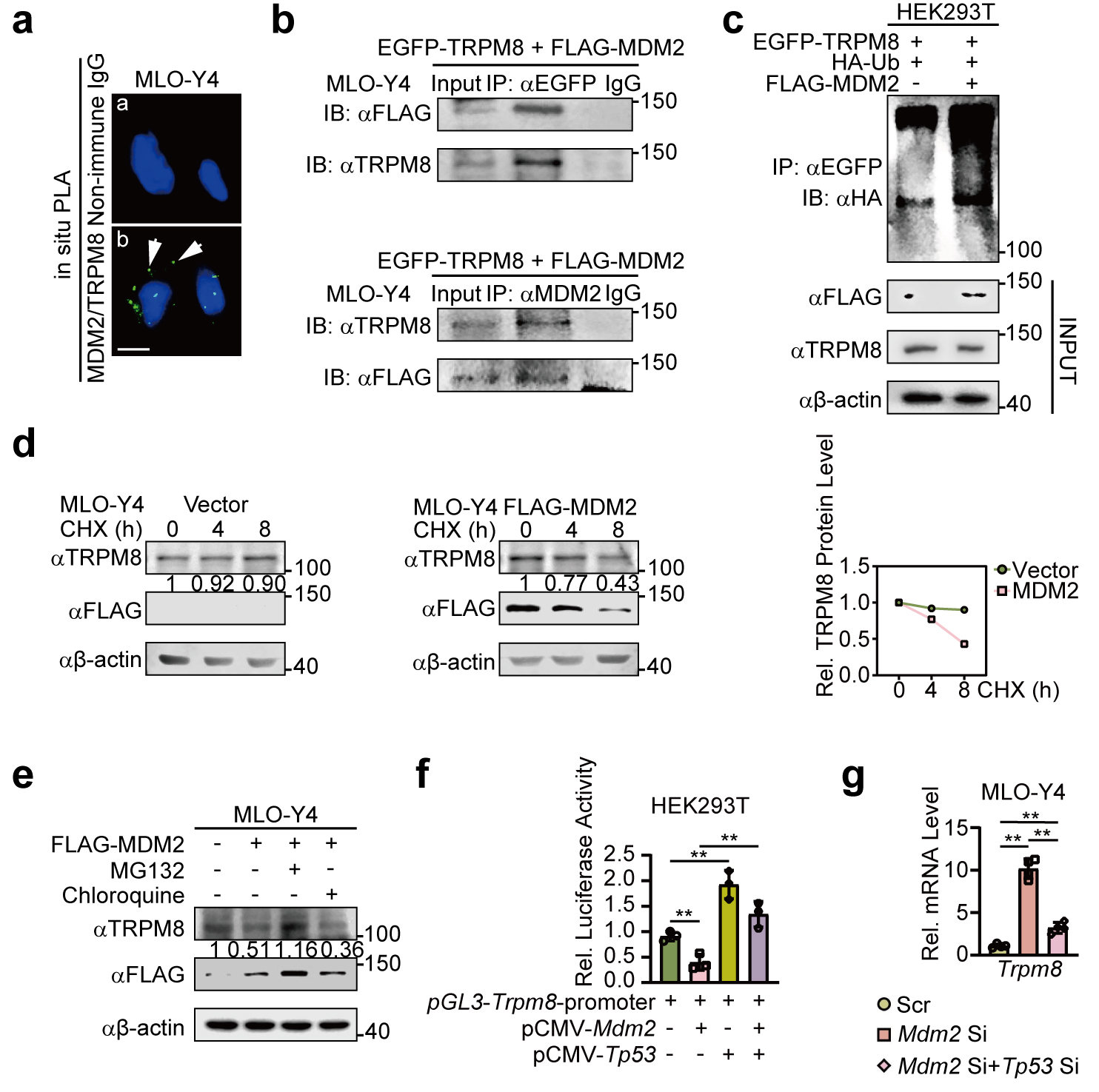

Supplement: Supplementary file 11 — Figure S10 [file 41413_2026_570_MOESM11_ESM.jpg]

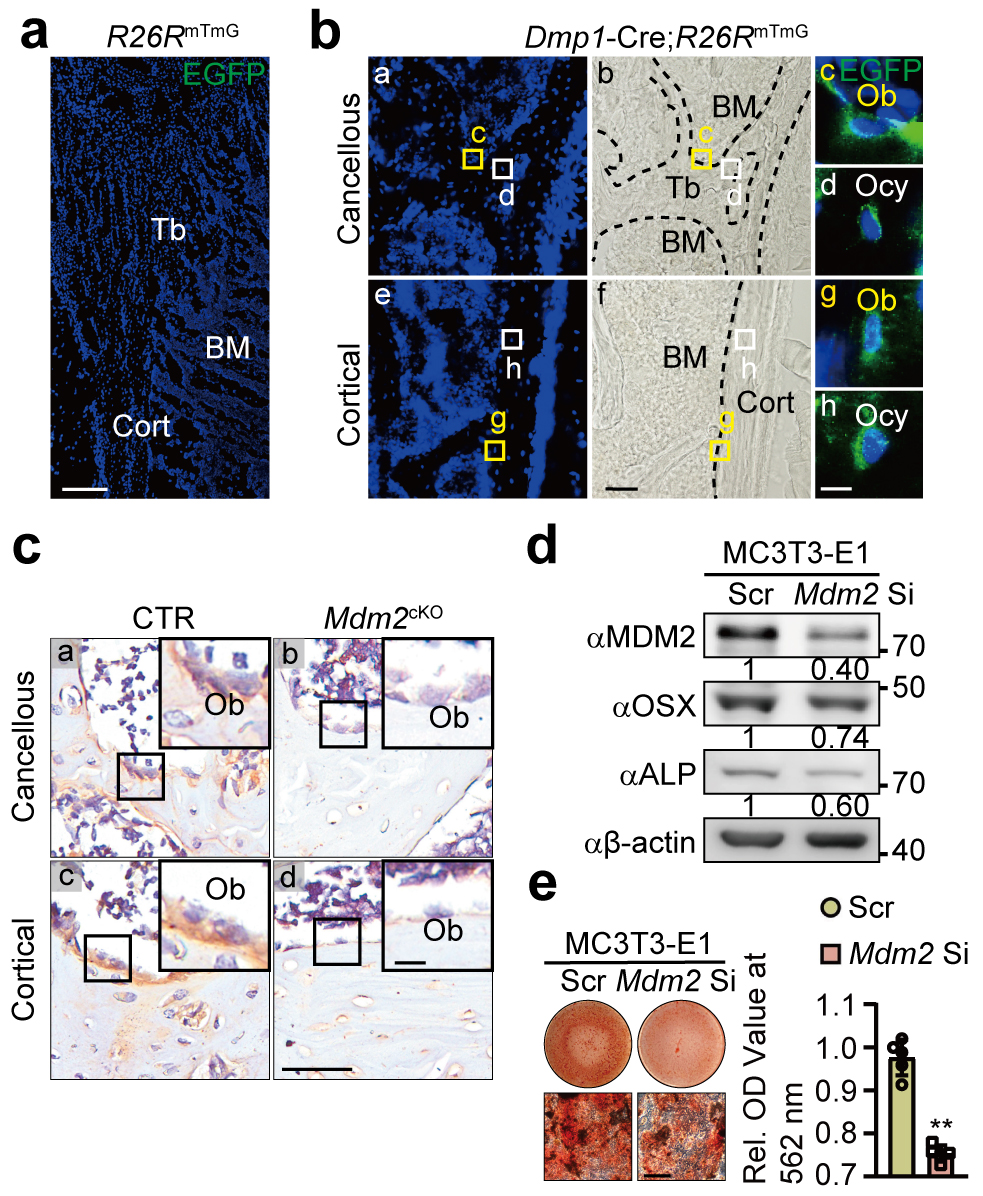

Supplement: Supplementary file 12 — Figure S11 [file 41413_2026_570_MOESM12_ESM.jpg]
